# Demystifying the Molecular Basis of Pyrazoloquinolinones Recognition at the Extracellular α1+/β3- Interface of the GABA_A_ Receptor by Molecular Modeling

**DOI:** 10.3389/fphar.2020.561834

**Published:** 2020-09-11

**Authors:** Natesh Singh, Bruno O. Villoutreix

**Affiliations:** ^1^Univ. Lille, INSERM, Institut Pasteur de Lille, U1177–Drugs and Molecules for Living Systems, Lille, France; ^2^Department of Pharmaceutical Chemistry, University of Vienna, Vienna, Austria

**Keywords:** GABA_A_ receptor, pyrazoloquinolinones, molecular docking, structure-activity relationships (SARs), MM-GBSA binding energies

## Abstract

GABA_A_ receptors are pentameric ligand-gated ion channels that serve as major inhibitory neurotransmitter receptors in the mammalian brain and the target of numerous clinically relevant drugs interacting with different ligand binding sites. Here, we report an in silico approach to investigate the binding of pyrazoloquinolinones (PQs) that mediate allosteric effects through the extracellular α+/β- interface of GABA_A_ receptors. First, we docked a potent prototype of PQs into the α1+/β3- site of a homology model of the human α1β3γ2 subtype of the GABA_A_ receptor. Next, for each docking pose, we computationally derived protein-ligand complexes for 18 PQ analogs with known experimental potency. Subsequently, binding energy was calculated for all complexes using the molecular mechanics-generalized Born surface area method. Finally, docking poses were quantitatively assessed in the light of experimental data to derive a binding hypothesis. Collectively, the results indicate that PQs at the α1+/β3- site likely exhibit a common binding mode that can be characterized by a hydrogen bond interaction with β3Q64 and hydrophobic interactions involving residues α1F99, β3Y62, β3M115, α1Y159, and α1Y209. Importantly, our results are in good agreement with the recently resolved cryo-Electron Microscopy structures of the human α1β3γ2 and α1β2γ2 subtypes of GABA_A_ receptors.

**Graphical Abstract:**
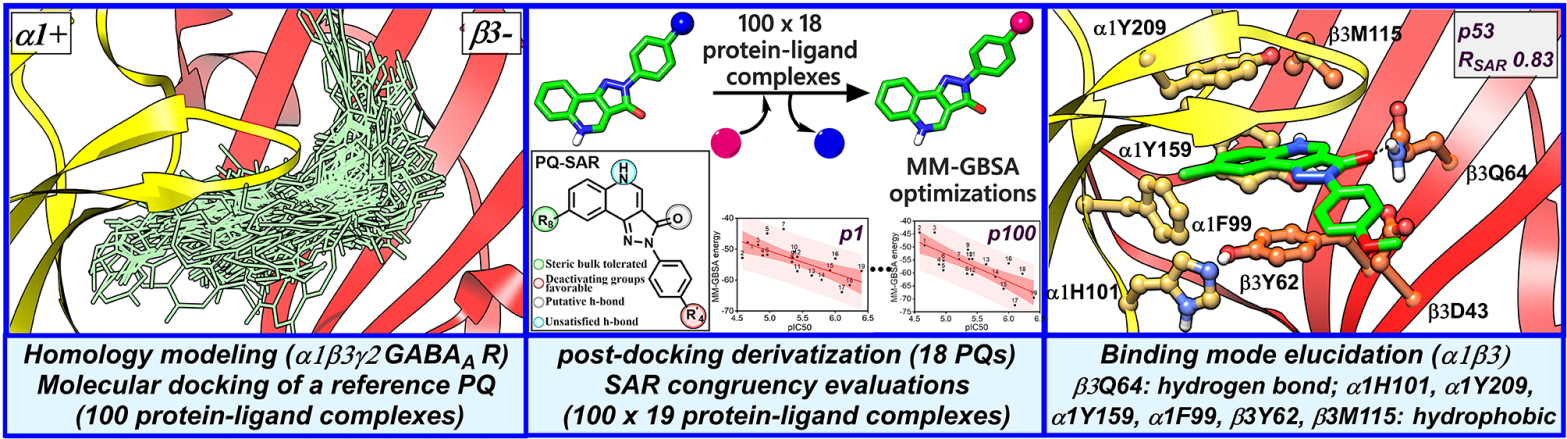


## Introduction

The structural elucidation of a ligand-receptor complex lays the foundation for efficient lead optimization cycles. However, the structural resolution of a protein complex can be time-consuming and very challenging, even more so for membrane proteins (Singh and Ecker, 2018; Scalise et al., 2020). In the absence of protein structure information, different methods can be used to identify putative hit compounds. Two prominent approaches include (1) structure-based design using homology modeling and molecular docking (Schmidt et al., 2014; Louet et al., 2017; Palazzolo et al., 2018; Singh et al., 2019a; Singh et al., 2019b; Singh et al., 2020c) and (2) ligand-based modeling (Chaput et al., 2020) using the structure-activity relationships (Kubinyi, 1998) (SARs) information derived from experimentally validated compound libraries. Notably, homology modeling, in conjunction with molecular docking, is widely used in virtual screening (Spyrakis and Cavasotto, 2015; Slater and Kontoyianni, 2019; Singh et al., 2020b). Investigating the possible ligand-binding modes facilitates hit-to-lead optimization and guides the rational design and synthesis of new chemical candidates with enhanced potency and selectivity for a target. Moreover, such knowledge can be taken into account to optimize the absorption, distribution, metabolism, and excretion (ADME) and toxicity parameters, such as solubility and metabolic stability, without disrupting essential ligand-receptor interactions (Greer et al., 1994; Maddaford, 2012). Also, this information can assist in identifying molecular determinants leading, for instance, to the agonist and antagonist behaviors of the ligands (Warne et al., 2011). However, studies have shown that docking programs are capable of reproducing the correct binding orientations, but the scoring functions often struggle to rank the correct orientations on top of the graded list (Siebert et al., 2018b). Hence, there is a need to identify new protocols and scoring techniques that increase the reliability of the binding hypotheses by assessing the congruency between the predicted and experimental binding affinity of the compounds.

**Figure 1:**
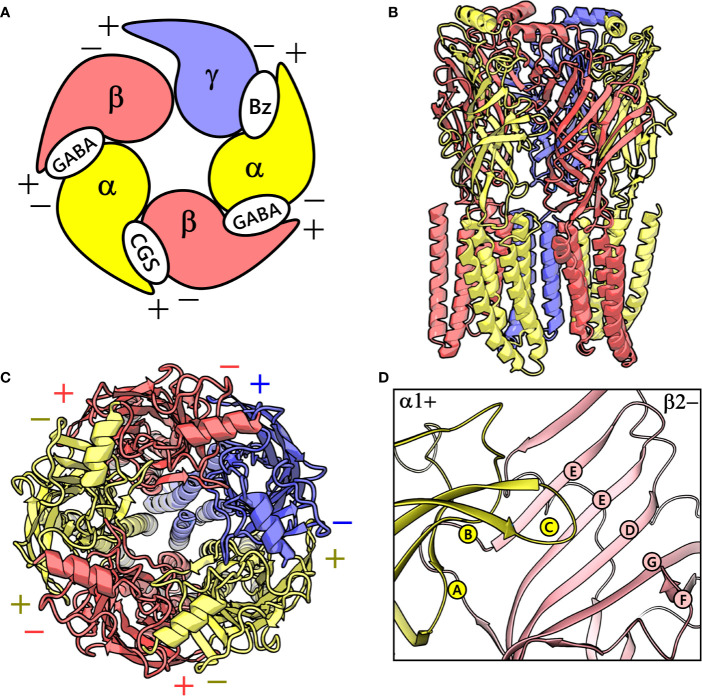
**(A)** A graphical depiction of the extracellular domain (ECD) of the GABA_A_ receptor. The different binding sites are indicated: the GABA binding site ‘GABA’, the high-affinity benzodiazepine binding site ‘Bz’, and the low-affinity ‘CGS’ site. **(B)** front view of the cryo-electron microscopy (cryo-EM) structure of the human α1β2γ2 GABA_A_ receptor (PDB ID: 6D6U). **(C)** The perpendicular top view of the structure. **(D)** Front view of the CGS site or α1+/β2- interface, which is characterized by the presence of loops A-C and D-G in the α1+ and β2- subunit, respectively. The α1, β3 and γ2 subunits are depicted in ribbon style and are colored yellow, red, and blue, respectively. **(B–D)** were prepared using PyMOL v. 1.8.6.2 (DeLano, 2008. Available at: https://www.pymol.org,” n.d.).

GABA_A_ receptors are ligand-gated ion channels that serve as essential molecular targets for several important clinical drugs like benzodiazepines, barbiturates, neuroactive steroids, anesthetics, and anticonvulsants (Sieghart, 2015). GABA_A_ receptors in mammals represent a heterogeneous cluster of pentameric receptors compiled from a pool of 19 potential subunits (α1-6, β1-3, γ1-3, δ, ϵ, θ, π, and ρ1-3) (Olsen and Sieghart, 2008). In the brain, the majority of the GABA_A_ receptors is composed of two α, two β, and one γ subunits (Olsen and Sieghart, 2008), and their arrangement can be described by topology β-α-γ-β-α (Tretter et al., 1997) (**Figure 1**), where each subunit interface, by convention, has a primary (+, plus) and a complementary (-, minus) side (Galzi and Changeux, 1994). γ-aminobutyric acid (GABA) binds to the extracellular part of the receptor at the interfaces between the α- and β+ subunits (**Figure 1A**). This leads to conformational changes that cause the channel to open and chloride anions to flow through (Jansen, 2019). Benzodiazepines *via* binding to an allosteric site located at the extracellular α+/γ- subunit interface mediate their anxiolytic, muscle-relaxant, sedative-hypnotic, and anticonvulsant effects (Sigel, 2002; Richter et al., 2012) (**Figure 1A**). Mutations affecting GABA_A_ receptors have been shown to cause neurological disorders such as epilepsy (Jansen, 2019).

The pyrazoloquinolinones (PQs) exhibit high potential as both non-sedative anxiolytics and as benzodiazepine antagonists (Savini et al., 2001; Vega Alanis et al., 2020) and, as such, represent interesting chemotypes. PQs exhibit features of both “continuous” and “discontinuous” SARs, depending on the corresponding substitution sites over the scaffold. A continuous SAR is described by a smooth activity hypersurface, where a clear trend in experimental activity could be detected upon systematic chemical changes, whereas a discontinuous SAR, in contrast, is depicted by a rugged landscape where slight structural modifications lead to drastic potency differences (Cruz-Monteagudo et al., 2014; Siebert et al., 2018b). Recent studies have demonstrated that in many subtypes of αβγ receptors, PQs exerts positive modulatory effects *via* an alternate allosteric binding site at the homologous α+/β- interface (**Figures 1A, D**) (Ramerstorfer et al., 2011). In contrast, the effects are antagonistic, i.e., flumazenil-like (Varagic et al., 2013a; Varagic et al., 2013b), when they bind at the high-affinity benzodiazepine site (α+/γ-) in most subtypes. Since a combined α- and β- isoform selectivity can be achieved, and binding is independent of additional subunits such as γ or δ, the α+/β- interface binding site is a potentially very attractive target for novel chemical probes (Simeone et al., 2017). Structural hypotheses of bound states would be helpful in developing more potent and possibly subtype-selective ligands. Recently, our colleagues elucidated the PQ binding mode at the benzodiazepine site (Siebert et al., 2018b) *via* a novel structure-based approach by utilizing ligand-based knowledge to frame a docking scoring function that assessed ligand binding poses for their congruency to recognized PQ-SAR. The important feature of this scoring scheme is the post-docking derivatization technique. This tool generates a congeneric series of protein-ligand complexes from the given set of docking poses through substituent placements, which can be used for rescoring (Zhenin et al., 2018; Rastelli and Pinzi, 2019; Singh et al., 2020a) and SAR congruency assessment.

**Figure 2:**
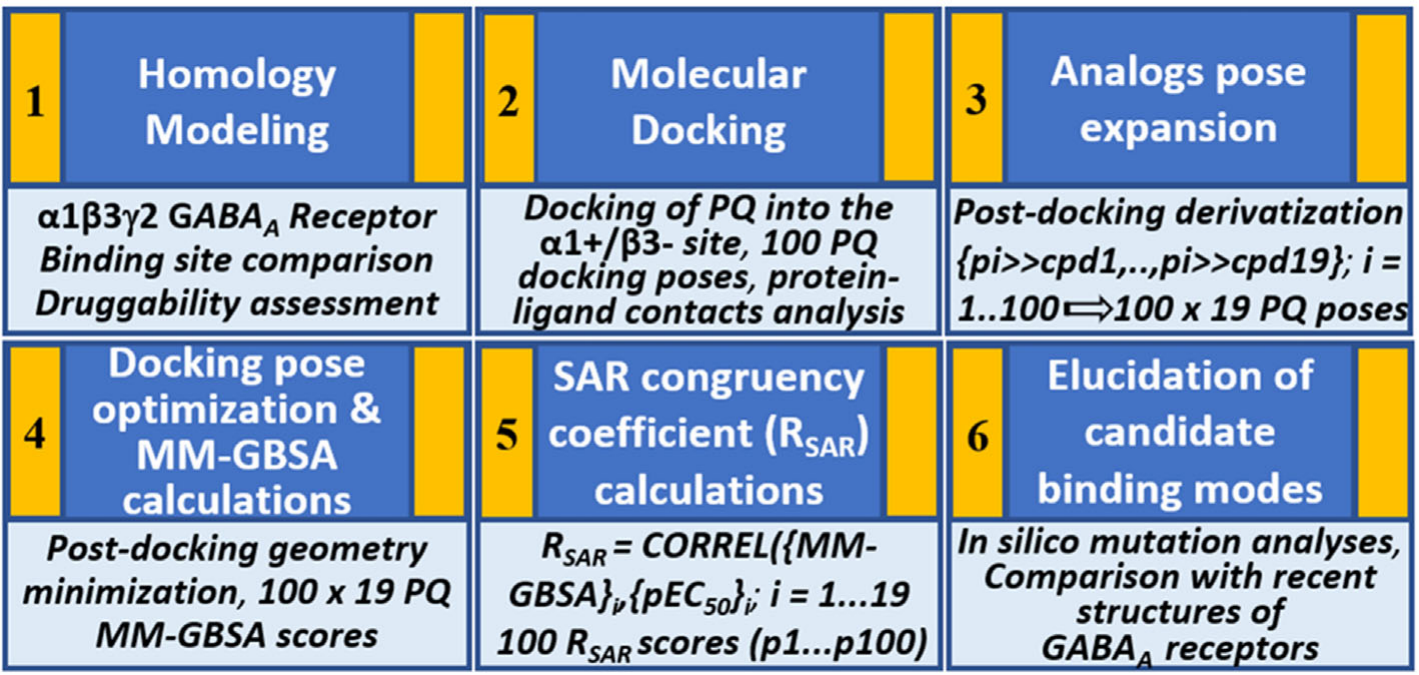
Structure-based workflow to identify the binding hypotheses for PQs at the α1+/β3- site step-by-step: (1) homology modeling of the human α1β2γ2 GABA_A_ receptor, (2) molecular docking of a reference PQ compound and interaction fingerprint analysis of the generated docking poses, (3) generation of the protein-ligand complexes of other PQ analogs using post-docking derivatization technique, (4) geometry optimization of the derivatized complexes and binding energy calculations, (5) SAR congruency coefficient calculations, (6) identification and characterization of the candidate binding modes.

We hypothesize that the PQs exhibit a common binding mode at the α+/β- site and that the correct orientation should be able to explain the inherent bioactivity trend and the experimental mutagenesis findings. Given the challenges associated with molecular docking and the concurrent availability of SAR data for PQs (Savini et al., 2001), we applied in this study, a structure-based protocol outlined in **Figure 2**. This approach integrates the ligand bioactivity information during the assessment process of the docking poses so as to define a binding hypothesis for PQs binding at the α1+/β3- interface. In the first step, a highly potent PQ, which has been extensively studied in multiple GABA_A_ subtypes, was docked into the α1+/β3- site of a homology model of the human α1β3γ2 subtype of the GABA_A_ receptor. This was followed by a pose expansion stage where we generated the protein-ligand complexes of 18 other PQ analogs from the recovered docking solutions by using a post-docking derivatization method. Subsequently, geometry minimization was performed, and binding affinity was estimated for all complexes using molecular mechanics-generalized Born surface area (MM-GBSA) approach (Genheden and Ryde, 2015) (**Figure 2**). The minimized complexes were then quantitatively evaluated by taking into consideration the experimental data through linear correlation calculations and then ranking the poses according to the SAR congruency score to determine the PQ top-ranked poses. Selected poses were optimized to investigate the previously reported 40-fold increase in potency of PQ ‘CGS-9895’ in the α1β3Q64A mutant (Siebert et al., 2018a). Finally, the results from the modeling and docking studies were compared with the newly solved cryo-Electron Microscopy (cryo-EM) structures of the human α1β2γ2 (PDB IDs: 6D6U, 6D6T) (Zhu et al., 2018) and the α1β3γ2 (PDB IDs: 6HUG, 6HUJ, 6HUK, 6HUO, 6HUP, 6I53) (Laverty et al., 2019; Masiulis et al., 2019) subtypes of GABA_A_ receptors. To the best of our knowledge, this is the first structure-based study devoted to the understanding of the α1β3 mediated ligand recognition, and the reported findings may facilitate the rational design and development of novel and selective chemical modulators of the α1β3 subunit interface.

## Results

### Homology Modeling of the α1β3γ2 Subtype of the GABA_A_ Receptor

**Figure 3:**
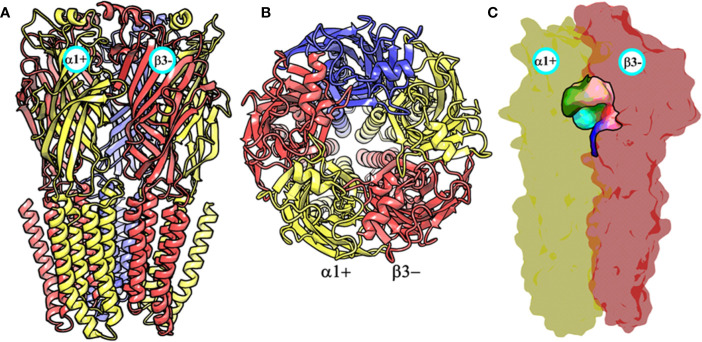
The front view **(A)** and the top view **(B)** of the homology model of the α1β3γ2 subtype of the GABA_A_ receptor. The α1, β3 and γ2 subunits are depicted in ribbon style and are colored yellow, red, and blue, respectively. The α1 and β3 subunits are indicated. **(C)** shows the α1+/β3- ligand-binding site. The binding site surface is colored according to the residue type, i.e., the green areas are hydrophobic, while the red, blue, and purple regions are hydrophilic. The α1 and β3 subunits are shown as molecular surfaces colored yellow and red, respectively. The figures were generated using PyMOL v. 1.8.6.2 (DeLano, 2008. Available at: https://www.pymol.org.,” n.d.).

At the time, when this study was started, the only GABA_A_ receptor structure that was available was a partial β3- subunit homopentamer (PDB ID: 4COF) (Miller and Aricescu, 2014). This structure was therefore used as a template for generating the structural models of the α1β3γ2 subtype using MODELLER (Sali and Blundell, 1993). The constructed models were assessed on the basis of normalized discrete optimized protein energy (z-DOPE) score. The top-ranked model had a DOPE score of -0.98, suggesting that approximately greater than 80% of its Cα atoms are predicted to be within 3.5 Å of their accurate positions (Eramian et al., 2008, p. 1), thus indicating a native-like structure (**Figure 3** and **Figure S1**). The overall quality of the model was then evaluated further using a Ramachandran plot after performing energy minimization with backbone atoms constrained. The PROCHECK (Laskowski et al., 1993; Laskowski et al., 2018) statistics showed that 94.4, 5.3, 0.3, and 0% of the residues, respectively, allocated as the “most favored,” “additionally allowed,” “generously allowed,” and “disallowed” regions (**Figure S2**). None of the residues located in generously or additionally allowed areas were in close proximity to the ligand-binding site. The model was also analyzed using the Profile-3D verify score (Eisenberg et al., 1997), which measures the compatibility score of each residue in the given 3D environment. The model returned a verify score of 676.33 that was close to the expected high score of 761.126, while the expected low score was 342.50. Models with a verify score between the reference values are considered sub-optimal and require refinement, while models with a value closer to the expected high score are likely to be correct. If the overall quality is lower than the expected low score, then the structure is almost certainly misfolded (Eisenberg et al., 1997). The model was lastly evaluated by measuring the root mean square deviation (RMSD) between its backbone atoms and those of the 4COF structure. The RMSD (0.33 Å) is very low, indicating further that the amino acids of the α1β3γ2 subtype can adequately accommodate in the template 3D structure (**Figure S3**). Overall, the structural analysis strongly indicates that the homology model of the α1β3γ2 subtype is accurate and can be used for docking studies.

### Binding Pocket Comparison Between α1β3 and α1γ2 GABA_A_ Receptor

**Figure 4:**
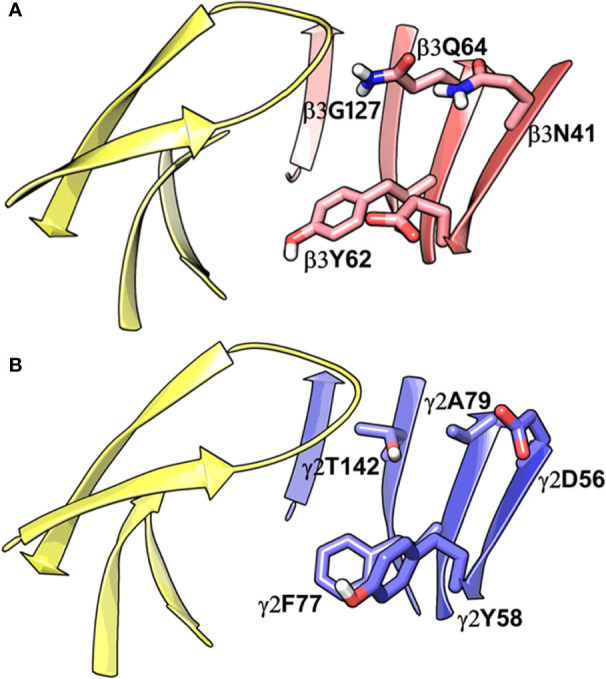
Comparison between the homology model of the α1β3γ2 subtype of the GABA_A_ receptor showing the α1+/β3- ligand-binding interface **(A)** and the cryo-EM structure of the α1β2γ2 GABA_A_ (PDB ID: 6D6U) receptor showing the α1+/γ2- interface **(B)**. The non-conserved residues β3N41, β3D43, β3Y62, β3Q64, and β3G127 on the β3-subunit and the corresponding beta residues γ2D56, γ2Y58, γ2A79, and γ2T142 are highlighted indicating the pocket differences. The α1, β3 and γ2 subunits are depicted in ribbon style and are colored yellow, red, and blue, respectively. The binding site residues are shown in stick style, and its carbon atoms are colored according to subunit.

**Figure 5:**
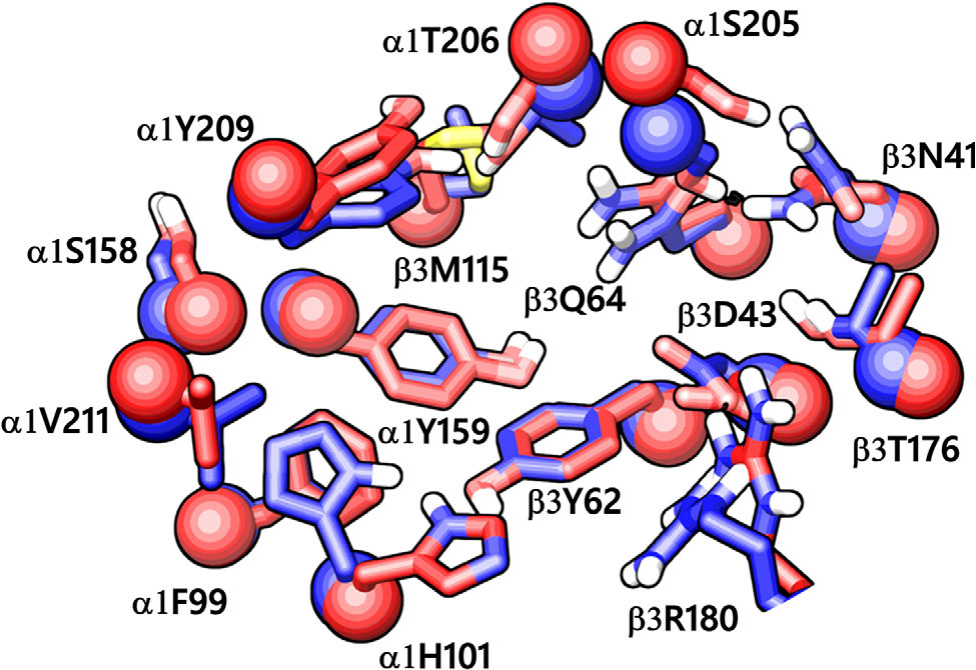
The α1+/β3- ligand-binding interface of the homology model (red) superposed to the corresponding site of the cryo-EM structure of α1β3γ2 GABA_A_ receptor (blue, PDB ID: 6HUJ). The Cα atoms and the side chains are shown in space-filling and stick style, respectively. The backbone RMSD between the two structures is 1.6 Å, and the alignment score is 0.1 suggesting good agreement with the experimental structure.

While writing this manuscript, several cryo-EM resolved heteropentameric structures of the human α1β2γ2 and α1β3γ2 subtypes of GABA_A_ receptors became available. These data allowed us to perform a post-hoc validation of the homology model, in which we carried out the docking simulations. The analysis of two homologous binding sites (α1+/β3- and α1+/γ2-) revealed that the hydrophobic residues γ2Y58 and γ2A79 at the α1+/γ2- interface are replaced by polar residues β2D43 and β2Q64 at the α1+/β3- interface. While the charged residue γ2D56 and the polar residue γ2T142 at the α1+/γ2- interface corresponds to polar β3N41 and hydrophobic β3G127 at the α1+/β3- interface (**Figure 4**). However, the active site residues belonging to the α1+ subunit are conserved at both interfaces, possibly indicating a similar α1 mediated interaction of the ligands. The backbone RMSD of the ECD between the two structures was 1.31 Å signifying the reliability of the homology model of the α1β3γ2 subtype. Whereas the Cα RMSD value for the residues enclosing the α1+/β3- and α1+/γ2- pocket was 0.95 Å indicating structurally similar ligand-binding sites. We also superposed the modeled α1+/β3- site with the corresponding site of the new α1β3γ2 structure (PDB ID: 6HUJ) (Masiulis et al., 2019). The alignment of the pockets displayed a low backbone RMSD of 1.6 Å (**Figure 5**), and it revealed similar binding orientation of the side chains of the residues emphasizing the high topological resemblance between the homology model and the experimental structure. The alignment showed that all the residues comprising the binding site in the homology model are identical to the cryo-EM structure, suggesting further that our homology model is accurate and appropriate for the docking studies. Next, we analyzed the binding site properties of α1+/β3- interface using SiteMap v3.4 (Schrödinger Release 2015-1, 2015c) (see Methods). The α1+/β3- site yielded a SiteScore of 1.11, a Dscore of 1.15, a volume of ∼335 Å^3^, and a total solvent accessible surface area of 752.25 Å^2^. The binding interface consists of 12.5% hydrophobic region, 54.5% hydrophilic region, and 33% mixed character region (**Figure S4**). The hydrophilic zone is partitioned into hydrogen bond donor and acceptor regions. The hydrogen bond donor region accounts for 52% of the hydrophilic region and the hydrogen bond acceptor region, 48% of the hydrophilic region. The hydrogen bond acceptor and donor regions refer to the degree that a well-structured ligand could interact with hydrogen bond donor and acceptor residues, respectively. The hydrophobic region contains residues like β3A45, β3Y62, α1F99, β3M115, β3L125, β3G127, β3L128, α1G157, α1Y159, α1V202, α1Y209, α1V210, and α1V211, whereas the hydrophilic region contains residues like β3D43, β3N41, β3Q64, α1H101, α1K155, α1S158, β3R169, β3T176, β3R180, α1S204, α1S205, and α1T206. Importantly, the recent structure (6HUJ) also exhibited similar binding site characteristics and returned a SiteScore and Dscore > 1, where a score higher than 1 (Halgren, 2009) suggests good druggability, yet again indicating the trustworthiness of the homology model and its appropriateness for structure-based investigations.

### PQ Dataset and Structure-Activity Relationship (SAR)

**Table 1** shows the experimentally measured data of PQs at the α1+/β3- and the α1+/γ2- interface retrieved from the scientific literature (Varagic et al., 2013b). For our protocol, we filtered those PQ compounds with reported pEC_50_ values against the α1+/β3- interface with the exception of meta-substituted (R^’^_3_) analogs (**Table 1**) to preserve the homogeneity of the data set. A QSAR study on the reported dataset revealed that lipophilic substituents at position R_8_ (ring A), as well as electron-withdrawing moieties at the R’_4_ position (ring D), are favorable for α1+/β3- potency. This is reflected by the substitution pattern found in analogs **16**, **17**, and **19**. Interestingly, in the α1γ2 PQ-SAR (Savini et al., 2001), the opposite is observed where electron-withdrawing groups dramatically reduce the potency. It seems that ring D in α1β3 is pointing toward an entirely different region than in α1γ2. The PQ data set has much higher variability in the R^’^_4_ position (nine diverse substitutions) than at the R_8_ position. At the R_8_ position, small hydrophobic moieties, noticeably chlorine atom, are favorable for affinity in α1β3 as compared to bulky substituents such as tert-butyl, indicating some steric hindrance at this position. Whereas, in α1γ2, the bulky substitutions are well tolerated at the R_8_ position. In α1γ2, the substitutions at the R_6_ position are sterically disallowed, and any substitution leads to a dramatic loss in affinity (Siebert et al., 2018b). Whereas the large tert-butyl substituent on position R_6_ (**7**) is tolerated at the α1+/β3- site. For a majority of the listed PQ analogs (**Table 1**), experimental data for the α1+/γ2- site is available (compounds **1**–**14**, **16**, and **18**). On average, the analogs exhibit four log units higher potency at the α1+/γ2- versus the α1+/β3- site. Next to this overall trend, we analyzed the relative potency change (Δα1β3 and Δα1γ2 in **Table 1**) of the analogs compared to the unsubstituted PQ scaffold (**1**) to assess substituent effects. In this analysis, the largest relative potency difference between the two binding sites was found for **7**, **12**, and **16**.

**Table 1:** The chemical structures of PQs and their biological activity values in pEC_50_ for the α1β3 and in pKi for the α1γ2 subtypes of GABA_A_ receptor.

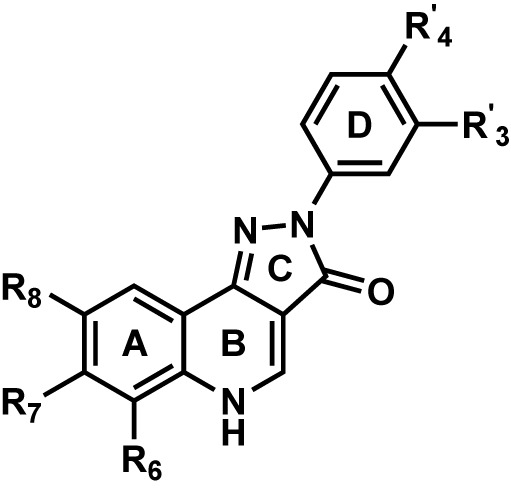										
S. No.	Cpd.	R_6_	R_7_	R_8_	R’_3_	R’_4_	α1β3 (pEC50)	α1γ2 (pKi)	Δ(α1γ3)	Δ(α1γ2)
**1**	CGS-8216	H	H	H	H	H	4.66	9.77	0	0
**2**	PZ-II-029	H	OMe	H	H	Ome	4.58	9.52	−0.08	-0.25
**3**	PWZ-009A1	H	Ome	H	H	H	4.82	8.89	0.16	-0.88
**4**	CGS 9895	H	H	H	H	Ome	4.89	9.49	0.23	-0.28
**5**	Xhe-III-24	H	H	tBu	H	F	4.95	9.6	0.29	-0.17
**6**	CGS 9896	H	H	H	H	Cl	4.96	9.3	0.3	-0.47
**7**	Xhe-II-087c	tBu	H	H	H	Br	5.21	7.47	0.55	-2.3
**8**	Xhe-II-006	H	H	tBu	H	Br	5.33	8.33	0.67	-1.44
**9**	PWZ-007A	H	H	Ome	H	H	5.35	10	0.69	0.23
**10**	Xhe-III-063	H	H	H	H	CCH	5.37	10.14	0.71	0.37
**11**	LAU 176	H	H	Ome	H	Ome	5.42	9.85	0.76	0.08
**12**	Xhe-II-17	H	H	tBu	H	CCH	5.42	8.48	0.76	-1.29
**13**	LAU 156	H	H	Cl	H	Me	5.64	10.3	0.98	0.53
**14**	PZ-II-028	H	H	Cl	H	Ome	5.79	9.7	1.13	-0.07
**15**	LAU 163	H	H	Cl	H	H	5.92	−	1.26	−
**16**	LAU 177	H	H	Ome	H	CN	6	9.12	1.34	-0.65
**17**	LAU 162	H	H	Cl	H	COOEt	6.1	−	1.44	−
**18**	LAU 206	H	H	Cl	H	NH2	6.22	9.92	1.56	0.15
**19**	LAU 161	H	H	Cl	H	CN	6.4	−	1.74	−

Analogs **2–19** are sorted according to α1β3 pEC_50_ values. The Δ(α1β3) and Δ(α1γ2) represents the difference in activity of the ligand with respect to the unsubstituted scaffold CGS-8216 (1).

### Molecular Docking of PZ-II-028

In this study, we applied a docking-based strategy that incorporates experimental activity data, as described in **Table 1**, to identify a common binding mode for PQs at the α1+/β3- interface. As a first step toward identifying a binding hypothesis, a potent ligand ‘PZ-II-028’ (**14**, **Table 1**) was docked into the α1+/β3- pocket. Since compound **14** has been extensively studied in different GABA_A_ receptor subtypes, and a large amount of experimental data is available for this ligand (Olsen and Sieghart, 2008; Varagic et al., 2013a; Varagic et al., 2013b; Mirheydari et al., 2014; Simeone et al., 2017; Treven et al., 2018); hence, this ligand serves as an excellent prototype (or reference ligand) for performing the docking studies. Molecular docking was performed using GOLD (Verdonk et al., 2003) with the flexible side chains option (see Methods). The distribution of 100 docking poses of compound **14** at the α1+/β3- is shown in **Figure S5**. To determine which molecular features are most relevant for binding, we performed structural interaction fingerprint (SIFt) (Deng et al., 2004; Singh et al., 2006) analysis of the docking poses of compound **14** using the cheminformatics utility of Schrödinger. This tool identifies the amino acid residues that show hydrogen bond or hydrophobic interactions with the docking poses. The SIFt analysis revealed that the docking poses were interacting with the amino acid residues of both α1+ and β3- subunits, situated throughout the pocket. The major residues involved in hydrogen bond interactions include α1Y159, α1S204, α1S205, β3N41, and β3Q64, whereas the residues α1Y209 and β3Y62 stimulated the binding through hydrophobic interactions (**Figure S6**).

### Post-Docking Derivatization and Binding Energy Calculations

Given the difficulties of scoring functions to correctly rank the ligands and to improve the quality of the poses obtained from docking into our homology model, we defined a scoring scheme that evaluates α1+/β3- PQ docking poses for its agreement with known PQ-SAR data. The scoring scheme for evaluating the 100 PZ-II-028 (p1-p100) poses consists of three steps: (i) analogs pose expansion using post-docking derivatization tool, (ii) energy minimization and binding energy calculations of the derivatized protein-ligand complexes, and (iii) SAR congruency assessment (i.e., calculation of correlation coefficient between the MM-GBSA scores and experimental data) and ranking of the poses according to the scores (see also **Figure 2**). The first preparatory step utilizes the previously published post-docking derivation tool (Siebert et al., 2018b) that results in the generation of the poses of related analogs (or ‘analog expansion’). Here, based on the 3D coordinates of every PZ-II-028 docking pose, an array of 18 ligand-receptor complexes for analogs **1**–**13**, and **15**–**19** (**Table 1**) is derived by adding substituents to the PQ scaffold of each docking pose of **14**. This step expanded the total protein-ligand binding poses to 1900 at the α1+/β3- from the first 100 docking poses of **14**. In the second step, the binding energy of all 1900 protein-ligand complexes was calculated by using the Prime MM-GBSA method implemented in Schrödinger. Briefly, this method utilizes the VSGB 2.0 implicit solvation model (Li et al., 2011) and OPLS-2005 force field (Banks et al., 2005) for the optimization of pose geometries and interactions (see Methods). The optimization step allowed to eliminate any potential ligand strain or steric clashes of the ligand atoms with the protein residues that might have developed after post-docking derivatization or due to the use of soft potentials while docking. In addition to the calculation of the MM-GBSA energy values, we also recorded the RMSD deviation of the PQ scaffold before and after the geometric optimization. In summary, the output of the analog expansion step is a set of 18 new energy-minimized ligand-receptor geometries and their corresponding MM-GBSA energy values and RMSD deviations.

### SAR Congruency Coefficient (R_SAR_) Calculation

**Figure 6:**
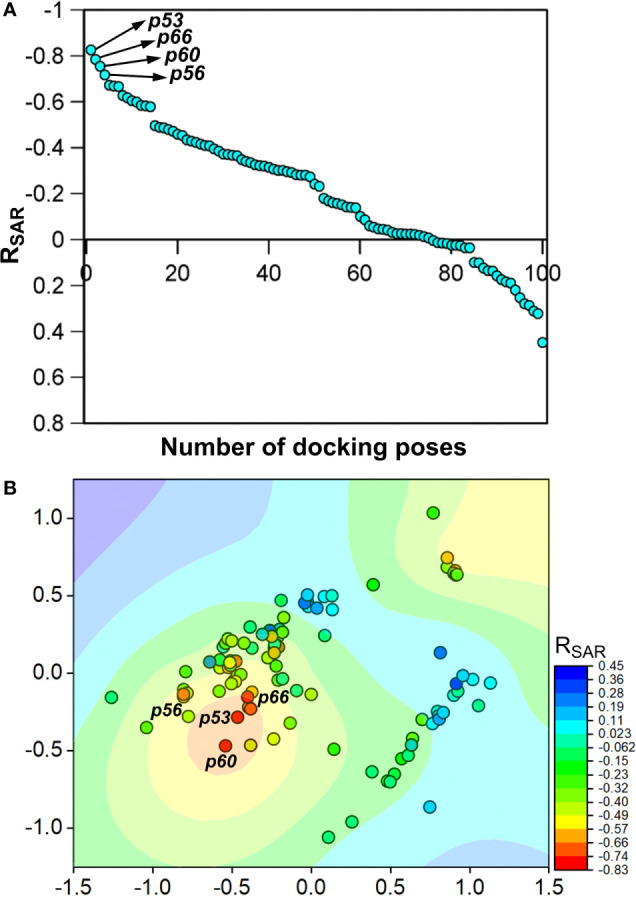
**(A)** The scree plot showing the SAR congruency score (R_SAR_) of the 100 docking poses. Four promising poses, p53, p66, p60, and p56, were identified from the correlation analysis. **(B)** Visualization of the geometric diversity of the optimized poses of **14** in the global pose space by using MDS. Each dot in the plot represents the docking pose, and its color indicates the R_SAR_ score for the analog series corresponding to the reference pose. The coefficient is decreasing from blue to red on the color scale.

**Figure 7:**
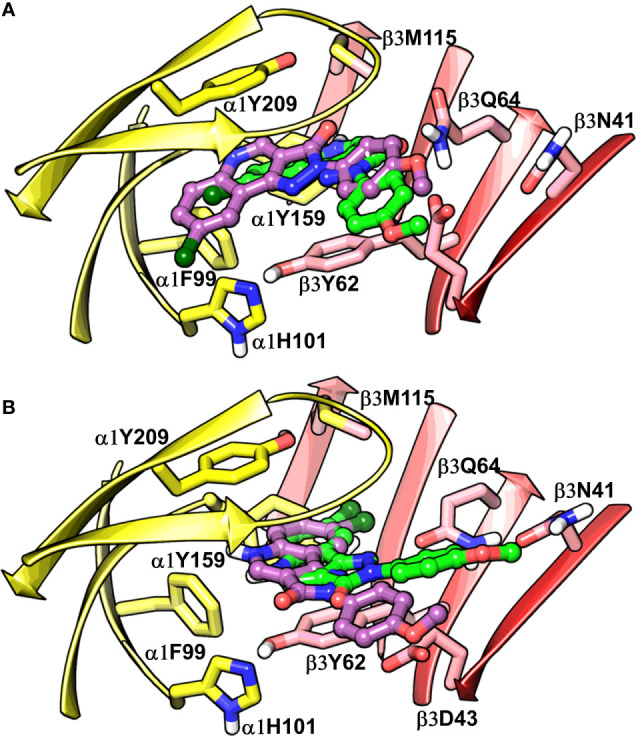
Best-predicted docking poses for compound **14** in α1β3. p53 (green) and p56 (violet) roughly corresponds to BM I **(A)**, while p60 (green) and p66 (violet) correspond to BM II **(B)**. In the figures, the ligand and the residues are depicted in stick-ball and stick style, respectively. The α1+ and β3- subunits are depicted in ribbon style and are colored yellow and red, respectively.

To assess the congruency of a PZ-II-028 docking pose and the resulting analog poses with existing experimental PQ SAR, we calculated the Pearson correlation coefficient between the predicted binding free energy (MM-GBSA) values of the “expanded analog set” and the corresponding bioactivity data (**Table 1**). Here, we refer to the correlation coefficient as the SAR congruency coefficient (R_SAR_) of a given docking pose. We calculated the R_SAR_ for the entire pose library (p1-p100) (**Figure S7**). To determine the most promising poses, we examined a scree plot (i.e., line plot) based on the R_SAR_ values (**Figure 6A**) and identified four poses, p53, p66, p60, and p56, that showed R_SAR_ of -0.83, -0.79, -0.75, and -0.72 and r^2^ of 0.68, 0.62, 0.57, and 0.51, respectively (**Figure 4A**). The Leave-one-out (LOO) cross-validation q^2^ follows the same trend as that of r^2^ e.g., p53 (0.60) < p66 (0.51) < p60 (0.47) < p56 (0.38) (**Table 2**). In addition, we performed Y-scrambling tests on these four docking poses (Rücker et al., 2007) using the QSPR/QSAR (Quantitative structure-property/activity relationship) “DEMOVA” package in R [“R Core Team (2018). R: A language and environment for statistical computing. R Foundation for Statistical Computing, Vienna, Austria. Available online at https://www.R-project.org/.,” n.d.]. To this aim, we shuffled the bioactivity values of the PQ dataset and calculated the r^2^_yscr_ and the LOO q^2^_yscr_. We obtained r^2^_ysc_ and q^2^_yscr_ values around 0 (P < 0.001) in all Y-scrambling experiments that were performed. Therefore, these results indicate that the statistical metrics obtained from SAR congruency calculations of the docking poses are not a consequence of spurious correlations. Despite the high RMSD among the top-ranked poses (> 2 Å) (**Table S1**), they can be broadly grouped into two geometrically different binding modes, BM I (p53 and p56) (**Figure 7A**) and BM II (p66 and p60) (**Figure 7B**) depending upon the orientation of the PQ ring. In BM I, the PQ ring is oriented in a manner such that the R_8_ substituent and the quinoline nitrogen is pointing toward the α1+ and β3- subunits, respectively, while in BM II the R_8_ substituent and the quinoline nitrogen (N5) are directed toward the β3- and α1+ subunit. The quantitative characteristics of the BM I and BM II poses obtained from the SAR congruency assessment and RMSD evaluations are shown in **Table 2**. While the regression plots between the predicted binding energy and pEC_50_ of PQs for the four poses are shown in **Figure S8**. Next, we utilized this R_SAR_ metric to visualize the geometrical variability of the 100 optimized reference poses of compound **14** from a global perspective by performing classical multidimensional scaling (MDS). The RMSD matrix of the 100 optimized poses of compound **14** representing high-dimensional conformational space or geometric heterogeneity served as an input for performing the dimensional reduction. The low-dimensional representation provides a meaningful description of the global pose space and enables the identification of docking poses that share a similar binding orientation. **Figure 6B** shows the MDS projection of the optimized poses for molecule **14** in the R_SAR_ landscape. Docking poses that are in close vicinity to each other share a similar binding orientation, whereas conformationally distinct or dissimilar poses are positioned distantly to each other. The MDS calculations further corroborate the geometric diversity among the top-ranked poses, which can be seen positioned distantly to each other in the plot. However, some less favorable poses are seen clustered near p56 and p66 that share similar binding orientation with low RMSD (<2 Å). The important limitation of the MDS in understanding pose diversity is that it fails to account for the binding characteristics of the poses that play an essential role in pose categorization and deciphering the crucial protein-ligand contacts. Overall, p53 (BM I) and p66 (BM II) were the two representative binding orientations of PQs identified from the R_SAR_ computations. Whereas p60 and p56 are of less interest due to their weak correlation with the PQ-SAR trend, and thus they were excluded from further analysis.

**Table 2:** Quantitative attributes of the top-ranked docking poses (p53, p66, p60, and p56) in terms of R_SAR_ score, root mean square error (RMSE), r^2^, leave-one-out (LOO) cross-validation q^2^, Y-scrambling r^2^_yscr_, Y-scrambling q^2^_yscr_, and RMSD’s with respect to the starting geometry.

Pose id	p53	p66	p60	p56
BM	BM I	BM II	BM II	BM I
SAR congruency coefficient (R_SAR_)	-0.83	-0.79	-0.75	-0.72
RMSE	0.3	0.33	0.35	0.37
r^2^	0.68	0.62	0.57	0.51
Leave-one-out cross-validation q^2^	0.60	0.51	0.47	0.38
Y-scrambling (1000 iterations) avg. r^2^_yscr_	0.05 ± 0.07	0.05 ± 0.07	0.04 ± 0.06	0.05 ± 0.07
Y-scrambling (1000 iterations) avg. q^2^_yscr_	0.05 ± 0.06	0.05 ± 0.07	0.05 ± 0.07	0.05 ± 0.06
p-value	P < 0.001	P < 0.001	P < 0.001	P < 0.001
Avgerage RMSD to starting geometry (Å)	0.77 ± - 0.67	1.02 ± 1.06	0.87 ± - 0.77	0.86 ± 1.10
Maximum RMSD to starting geometry (Å)	2.45	3.22	2.84	3.39

The reported p-values indicate the statistical significance of the correlation calculations (α = 0.05).

**Table 3:** Ligand interactions with the protein residues observed in the respective binding mode as determined by the ‘protein-ligand interaction’ tool implemented in Maestro.

Subtype	Cpd	α1H101	α1S204	α1Y209	β3Q64	β3Y62/γ2F77	α1Y159
α1β3 (BM I)	**14**	Hydrophobic	–	Hydrophobic	h-bond	Hydrophobic	Hydrophobic
α1β3 (BM II)	**14**	Hydrophobic	–	π-π	–	π-π	Hydrophobic
α1γ2	**1**	π-π	–	π-π	–	Hydrophobic	Backbone h-bond, hydrophobic
α1γ2	Flumazenil	h-bond, hydrophobic	–	π-π	–	π-π	Hydrophobic
α1β3Q64A	**4**	π-π	–	π-π	–	Hydrophobic	Backbone h-bond, hydrophobic
α1β1	**14**	π-π	h-bond	π-π	h-bond (b1Q64)	Hydrophobic (b1Y62)	Hydrophobic

The compound ids are indicated in bold as per **Table 1**.

### Characterization of the Candidate Binding Modes (BM I and BM II) at the α1+/β3- Interface

**Figure 8:**
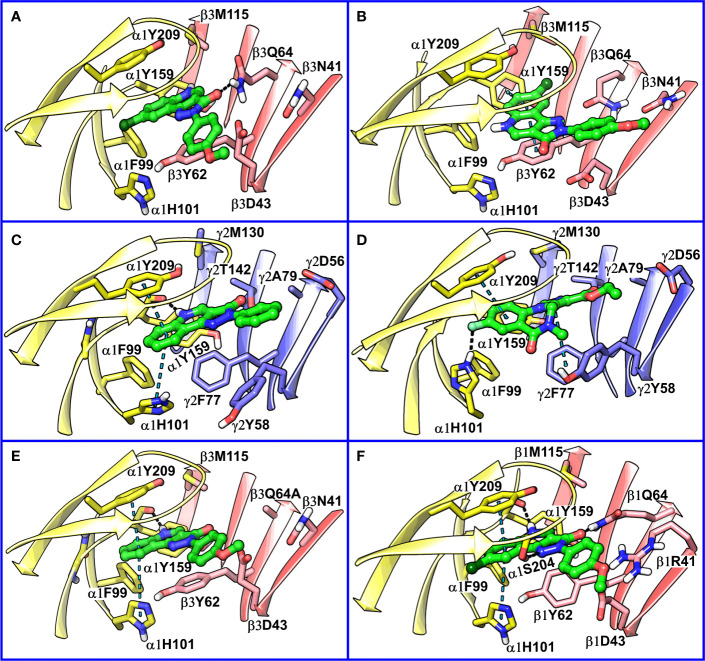
**(A)** predicted binding mode of compound **14** (p53, BM I) (green) at the α1+/β3- interface; binding energy: -48.3 kcal mol^-1^ **(B)** predicted binding mode of **14** (p66, BM II) (green) at the α1+/β3- interface; binding energy: -61.4 kcal mol^-1^ **(C)** predicted binding mode of **2** (green) at the α1+/γ2- site. **(D)** binding mode of Flumazenil (green) at the α1+/γ2- interface of the α1β2γ2 subtype of the GABA_A_ receptor (PDB ID: 6D6U). **(E)** optimized binding mode of **4** (green) in the α1β364A mutant; binding energy: -48.2 kcal mol^-1^ (β3Q64A) and -44.0 kcal mol^-1^ (β3Q64) **(F)** predicted binding mode of **14** (green) at the α1+/β1- interface; binding energy: -73.3 kcal mol^-1^. In the figures, the ligand and the residues are depicted in stick-ball and stick style, respectively; the α1+, β3-, and γ2- subunits are depicted in ribbon style and are colored yellow, red, and blue, respectively. The black and blue dotted lines in the binding modes indicate hydrogen bond and π-π interactions, respectively.

The visual inspection of the best performing BM I pose, p53 for the PQ **14** revealed that the aromatic pyrazoloquinoline scaffold is deeply buried in a sub-pocket formed by hydrophobic and aromatic residues (α1F99, α1H101, α1V202, α1Y209, and β3Y62) (**Figures 7A** and **8A**). In this pose, we observed major hydrophobic interactions of the fused ring system with the residues α1Y209 and/or β3Y62. Also, PQ is engaged in favorable van der Waals contacts with the protein residues α1Y159, α1F99, and β3M115. In contrast to the favorable orientation of the pyrazoloquinoline ring, the pending phenyl moiety (ring D) is only poorly bound, positioned unfavorably close to the acidic residue β3D43, and exposed to the solvent. This orientation of the rings is consistent with the observation of Varagic et al. (2013b) who proposed that upon binding, the ring D and the R_4_^’^ substituent of PQ are located in a hydrophilic environment, whereas the R_8_ substituent extends into a hydrophobic pocket. Besides the hydrophobic interactions, PQ is engaged in electrostatic interactions as well. The methoxy group at the R^’^_4_ position of the phenyl ring is donating a hydrogen bond to the residue β3R180, while the carbonyl group is accepting a hydrogen bond from β3Q64. The quinolone and pyrazole nitrogens are not engaged in any polar contacts. Docking pose p53, in addition, supports the SAR trend where the presence of strong deactivating groups at the R’_4_ position in highly active PQs (pEC_50_ > 6) such as **16**, **17**, and **19** seems to reduce the electron density over the pending phenyl ring, thus alleviating the unfavorable electrostatic interaction with β3D43 and eventually resulting in potency gain. While the activating effects of the amino group at the R’_4_ position in **18** seem to be compensated through a strong hydrogen bond interaction with the side chain of β3T176 (**Figure S9**) that might explain its high potency. The low activities of PQs **1**–**6** (pEC_50_ between 4 and 5) can be attributed to the lack of lipophilic substituent at the R_8_ position that allows for favorable interactions with the hydrophobic subpocket. However, PQ **5** is comparatively better than the other members owing to the presence of a hydrophobic tert-butyl group at the R_8_ position. The PQs **7**–**15** are moderately active (pEC_50_ between 5 and 6), and maximum members of this group, except **7** and **10**, have a lipophilic moiety attached at the R_8_ position, which is engaged in hydrophobic interactions involving α1H101, α1V202, and α1Y209. These findings are consistent with those of Varagic and coworkers who showed that the electron-withdrawing substituents on rings A and D, as well as lipophilic R_8_ and hydrophilic R_4_^’^ substituents, are beneficial for high potency (Varagic et al., 2013b). Interestingly, the favorable effect of electron-withdrawing moieties at the R’_4_ position in α1+/β3- (Varagic et al., 2013b) is inverted in the α1+/γ2- site (Savini et al., 2001). The two outliers of p53 with the poorest prediction (i.e., showing high residuals) were the PQs **11** and **18**. Removing these two PQs from the dataset and re-assessing the SAR congruency increased the R_SAR_ score from 0.83 to 0.9 and r^2^ from 0.68 to 0.79 (**Figure S10**). This improvement in results further indicates that p53 can very well explain the variation in the bioactivity of the PQs. In BM II (p66) (**Figures 7B** and **8B**) the pyrazoloquinolinone scaffold is flipped by ~180° with respect to BM I, resulting in an orientation where the chlorine atom at the R_8_ position and the quinoline nitrogen are directed toward the β3- and α1+ subunit, respectively. Likewise to BM I, the hydrophobic pending phenyl ring is positioned unfavorably close to the acidic residue β3D43. In contrast to BM I, no hydrogen bond interactions between **14** and the receptor were observed. The protein-ligand contacts of the poses are enumerated in **Table 3**.

### Comparison of α1+/β3- Binding Mode With α1+/γ2- Binding Mode

Recently, our coworkers elucidated the binding mode of PQ at the high affinity α1+/γ2- site (**Figure 8C**). Interestingly, BM I in α1+/β3- (**Figure 8A**) shows a qualitatively similar binding orientation to the PQ scaffold as in the α1+/γ2- site. In both orientations, the quinoline ring is located underneath loop C and shows hydrophobic and/or pi-pi interactions interaction with α1Y209, α1F99, and β3Y62 or γ2F77, respectively (**Table 3**). In contrast to the α1+/γ2- site, the quinoline nitrogen in α1+/β3- does not display hydrogen bond interaction with the backbone of α1Y159. The altered steric and electrostatic pocket requirements shaped by β3Q64 and β3D43 might push the quinoline scaffold in a position that impedes the quinoline α1Y159 interaction in the α1+/β3- site. The significant difference in the PQ interaction profile between the α1+/β3- and the α1+/γ2- site is understood by the orientation of the pending phenyl moiety. While, the moiety is placed disfavorably in the α1+/β3- site close to the acidic β3D43 residue, it shows favorable hydrophobic interactions with the equivalent residue γ2Y58 in the γ2 subunit. The absence of crucial interactions in the α1+/β3- in comparison to the α1+/γ2- interface aligns with the experimental finding of 4 log potency differences in the two subunits.

### Comparison of α1β3 CGS-Binding Modes, BM I and II, With Flumazenil Structure

We further compared BM I and BM II of **14** at the α1+/β3- site with the cryo-EM structure of human α1β2γ2 GABA_A_ receptor (PDB ID: 6D6U) (Zhu et al., 2018) complexed with flumazenil (Ro15-1788) at the benzodiazepine site (α1+/γ2-). In the solved structure, the imidazobenzodiazepine ring of flumazenil is oriented parallel to loop C, with fluorine at the 7^th^ position and carboxylate group at the 3^’^ position is accepting a hydrogen bond from the side chain of α1H102 and γ2T142, respectively (**Figure 8D**). The terminal ethyl group is extending toward the solvent between the tip of loop C and loop F. The imidazobenzodiazepine ring is involved in two π-π interactions involving residues α1Y210 and γ2F77. The residues α1Y159, γ2Y58, and γ2A79 are further contributing to the binding of flumazenil *via* favorable hydrophobic interactions (**Figure 8D**). We then superposed BM I to the binding orientation of flumazenil (**Figure S11**). The alignment revealed that the PQ ring is overlapping with the imidazobenzodiazepine ring of flumazenil. Whereas the pending phenyl ring of **14** and the terminal ethyl group of flumazenil are oriented away from each other and are solvent-exposed at both interfaces (**Figure S11**). Interestingly, the halogen atoms, Cl and F, in both structures are pointing toward the hydrophobic region of the α1+ subunit. The distance between the center of mass (COM) of the two ligands is 0.84 Å, indicating high commonality in the binding orientation, but with different binding strengths, at the two homologous sites of the GABA_A_ receptor. While in the case of BM II, the distance between the COM of the ligands, **14** and flumazenil, is 1.81 Å (**Figure S12**), indicating that BM II differs from the binding orientation of flumazenil. Also, due to the flipping of the fused ring in BM II, the quinoline nitrogen is occupying a position equivalent to the fluorine of flumazenil, which is in contradiction to BM I-flumazenil superposition, where the two halogen atoms are overlapping with each other. Based on the analysis of two distinct BMs with the flumazenil structure, it can be inferred that BM I is indeed more reliable than BM II to account for the binding of PQs. This set of results is further consistent with the new structures of the α1β3γ2 subtype (Masiulis et al., 2019) complexed with diazepam or alprazolam at the α1+/γ2- site, in which we observe a tight ligand volume overlap and a common interaction profile hallmarked by the ligand interactions with the residues α1H101, α1Y209, and α1Y159. In summary, the recent structures strengthen the BM I-like PQ binding orientation at the α1+/β3- site.

### Analysis of α1β3Q64 Mutation

Siebert et al. reported a 40-fold increase in potency of CGS-9895 (**4**) in the α1β3Q64A mutant (Siebert et al., 2018a). At first sight, this is inconsistent with BM I in which β3Q64 acts as a hydrogen bond donor to the carbonyl-oxygen of the PQ scaffold, and the mutation would lead to the abolishment of this interaction. On the other hand, we observed that the large β3Q64 residue is pushing the PQ-scaffold away from a high affinity α1+/γ2- orientation. To computationally assess the effect of the mutant on our BM I orientation (p53), we converted the β3Q64 residue to its gamma analog alanine in the binding pose of **4** and **14** and then conducted in-situ ligand minimization followed by binding free energy calculations. The geometry optimization was performed using the OPLS-2005 force field and the Truncated Newton Conjugate Gradient (TNCG) minimization algorithm (see Methods). The RMSD of the poses of **4** and **14** between the wild-type and the mutant protein was 1.5 and 1.48 Å, respectively, indicating a considerable change in the binding orientation after optimization. Interestingly, the mutation from β3Q64 to its gamma analog and subsequent energy minimization resulted in a ligand orientation that displays a more “α1γ2”-like interaction, i.e., two π-π interactions of the quinoline ring with α1Y209 and α1H101 and one backbone hydrogen bond of the quinoline nitrogen with α1Y159 (**Figure 8E** and **Figure S13**). Also, the binding energy of the optimized poses in the α1β3Q64A mutant was higher as compared to the α1β3Q64 wild-type pose. We reason that the mutation of β3Q64 to alanine increases the ligand-binding surface area that allows the ligand to readapt in an orientation where it can engage in favorable interactions with the binding site residues. Overall, the results obtained here are consistent with the experimental findings of the increased potency of **4** in the α1β3Q64A mutant.

### Extrapolation of BM I to the α1+/β1- Site

The amino acid residues at both α1+/β1- and α1+/β3- interfaces are highly conserved and only show differences in position 41 and 180 of the β3 subunit (**Figure S14**). The residue β3N41 at the α1+/β3- correspond to β1R41 at the α1+/β1-, whereas β3R180 at the α1+/β3- is equivalent to β1K180 at the α1+/β1-. Despite these small differences in the pocket, the pEC_50_ of **14** is approximately 30 times higher in the α1+/β1- compared to the α1+/β3- interface (Simeone et al., 2017). To analyze this experimental finding in the context of our BM I pose (p53), we performed molecular docking of **14** at the α1+/β1- site using an α1β1γ2 GABA_A_ homology model (**Figures S1** and **S15**) and generated 100 docking poses (**Figure S16**). From the optimized docking poses of **14**, we calculated the RMSD difference to the best performing α1+/β3- BM I pose (p53). This led to the identification of a docking pose that exhibited minimum RMSD with p53 (2.2 Å) and showed higher binding energy compared to BM I in α1β3 (**Figure 8F**). In addition, this pose displayed a good overlap with the binding mode reported by Siebert et al. (2018b) and the new GABA_A_ structures. In the docking pose, the quinoline ring is engaged in two π-π interactions involving residues α1Y209 and α1H101, and the quinoline nitrogen atom is donating a hydrogen bond to the backbone of α1Y159 (**Figure 8F**). In addition, the pyrazolone ring (ring C) is engaged in two hydrogen bond interactions with the side chain of the residues α1S204 and β1Q64. The residues α1F99 and β1Y62 are additionally mediating the binding of the ligand through hydrophobic interactions. Analogously to the α1+/β3- interface, the pending phenyl ring is located unfavorably close to β1D43 and is solvent-exposed. However, the negative effects of the charged β1D43 are likely diminished by a salt-bridge with the aforementioned β1R41 residue. Overall, the results achieved are consistent with the increased biological activity of PZ-II-028 for the α1β1 subtype.

## Discussion

The identification of a ligand-receptor complex can significantly assist drug design programs through iterative multiparameter ligand optimization steps. However, the experimental structural elucidation of protein-ligand complexes is a multifaceted and time-consuming process, and it is often unfeasible for many membrane-bound protein targets. Here, homology modeling of a target protein in combination with molecular docking serves as an essential computational tool that can generate reasonable binding hypotheses (Miteva et al., 2005; Villoutreix et al., 2013; Ishoey et al., 2018; Lagarde et al., 2019; Singh et al., 2019b).

PQs exerts modulatory effects similar to benzodiazepines *via* the extracellular α1+/β1- or α1+/β3- ligand-binding site of the GABA_A_ receptors. However, the molecular basis of interaction at the α+/β- interface has remained elusive so far. To strengthen the reliability of the selection of a docking pose for the prediction of binding hypothesis, we herein developed an automatized routine that was applied to a set of molecules exhibiting a distinct SAR for the α1+/β3- subtype of the GABA_A_ receptor. We first docked a potent PQ **14** compound into the α1+/β3- pocket and generated 100 diverse docking poses. To evaluate these different binding orientations, we derived protein-ligand complexes, *via* substituent placements, for 18 other PQs, **1**–**13** and **15**–**19**, using the coordinates of each docking pose of **14**. This was followed by MM-GBSA refinement to optimize the derivatized complexes and determine the protein-ligand binding energy. Subsequently, the optimized protein-ligand complexes were quantitatively evaluated by means of R_SAR_ score between the predicted binding energy and biological activity data to assess the congruence between the analog placement and the PQ-SAR.

Our SAR guided docking pose estimation led to the identification of one favorable binding mode (BM I, p53) (**Figure 8A**) that is harmonious with the PQ-SAR as reflected by a maximum negative R_SAR_ score of -0.83 and a maximum r^2^ of 0.67. Also, BM I showed a low average and maximum RMSD of 0.75 Å and 2.45 Å to the reference pose, indicating a minimum disparity in the binding orientation among the PQ analogs poses. To evaluate the 40-fold increase in potency of **4** in the α1β3Q64A mutant (Siebert et al., 2018a), we performed an in-situ ligand minimization of BM I of **4** with β3Q64 mutated to alanine followed by binding free energy calculations. The optimized BM I revealed two π-π interactions with the residues α1H101 and α1Y209, and a backbone hydrogen bond interaction with the residue α1Y159, that might elucidate the high potency of **4** in the mutant. Importantly, these were the same set of interactions that were previously described by Siebert et al. for PQ CGS-9895 at the α1+/γ2- interface (Siebert et al., 2018b) indicating strong coherence between the two BMs at the homologous ligand binding interfaces. In addition, BM I showed good overlap to the binding orientation of flumazenil at the α1+/γ2- interface of the α1β2γ2 GABA_A_ receptor further signifying the reliability of BM I. A second, moderately performing binding mode, BM II, pose 66, (R_SAR_ score: -0.79, r^2^: 0.61) (**Figure 8B**) was also identified that showed a high average and maximum RMSD of 1.02 and 3.22 Å to the reference pose indicating greater variability in the orientation of the poses. Furthermore, BM II showed poor overlap with the binding mode reported by Siebert et al. and the flumazenil structure indicating that, indeed, BM I is more promising than BM II to account for the binding of PQs at the α1+/β3- interface.

Taken together, our docking protocol led to the detection of one convincing binding mode (BM I), providing a structural rationale for the PQ-SAR (**Table 1**) in α1β3. In BM I, the fused ring system show hydrophobic interactions with α1Y159, α1F99, β3Y62, and β3M115, while the pending phenyl ring D is extending toward the solvent, which is consistent with the findings of Varagic et al. (2013b). The ring C is involved in a strong hydrogen bond interaction with β3Q64 that appears to be the main force driving the affinity apart from the contributions through hydrophobic interactions (**Figure 8A**). Notably, BM I showed the absence of backbone hydrogen bond interaction of the quinoline nitrogen which seems to be an essential interaction to gain affinity as suggested by Siebert et al. (2018b) In combination with the loss of backbone interaction and diminished hydrophobic interactions, this altogether explains the overall low affinity of PQs at the α1+/β3- interface in comparison to the α1+/γ2- interface. Importantly, β3D43 is revealed as a crucial residue hindering the binding of PQs at the α1+/β3- interface due to the electrostatic repulsion between the carboxyl group of D43 and the electron-rich areas of the ligand. The presence of strong electron-withdrawing groups at the R^’^_4_ position in PQs, **16**, **17**, and **19**, seems to reduce the electron density over the ring D, thus decreasing the degree of the electrostatic clash with β3D43. Additionally, these PQs are enabled with a lipophilic group at the R_8_ position resulting in strong interaction with the hydrophobic subpocket. This might explain their high affinity compared to the reduced activity of PQs **1**–**6** and moderate activity of PQs **7**–**15**, which have either strong or moderately activating groups substituted at the R^’^_4_ position. The importance of β3Q64 for PQ binding was revealed in the β3Q64A mutant, which led to a 40-fold increase in potency for **4**. Our in silico mutagenesis and energy calculations showed that the mutation of β3Q64 to alanine results in the increase of binding surface area that allows the ligand to accommodate in an energetically favorable orientation, which might explain the high affinity of compound **4** in the mutant protein. The optimized mutant pose and associated interactions were found to be in good agreement with the binding features described by Siebert et al. (2018b), which reinforces the reliability of BM 1. The high affinity of PQs for the α1β1 subtype compared to α1β3 can be explained by the presence of a positively charged residue β1R41 that allows for electrostatic interactions such as π-π or a cation-π interaction with the ring D of the ligand and possible reduction in electrostatic repulsion *via* a salt-bridge interaction with β1D43. Whereas in the α1β3 subtype, no such interactions were observed that have a neutral N41 in the same position. Also, the docking pose in α1β1 shows a conserved hydrogen bond interaction of the quinoline nitrogen with the backbone of α1Y159 and two π-π interactions involving α1H101 and α1Y209, that is, consistent with the findings reported by Siebert et al., (2018b). Despite the good agreement of BM I with the previous studies and the recent GABA_A_ structures, there is a need for structures with ligand bound to the α+/β- site in order to understand the binding orientation better. Moreover, this would allow benchmarking of docking studies against these structures, which definitely would increase the validity of the binding hypotheses retrieved.

However, next to SAR availability, the applicability of our protocol strongly depends on the characteristics and the quality of the underlying SAR. Incongruent SAR patterns, as well as an inadequate SAR-hypersurface, may be considered as limiting factors that impede the proposed approach. For example, a flat SAR without any discontinuity would not carry any discriminative potential for pose prioritization (Siebert et al., 2018b). Here, the calculation of the R_SAR_ scores might provide a quick suitability assessment. In terms of target space, we believe that due to the rigorousness of the post-docking derivatization and subsequent SAR congruency assessments, our protocol might be more applicable to proteins accommodating rather tight and narrow binding pockets. Hence, in addition to R_SAR_ calculations, B-factors analysis (Vihinen et al., 1994), binding site analysis, and techniques to assess protein flexibility such as molecular dynamics (MD) simulations may provide estimates for the suitability of our protocol at a given context.

The improvement over the previously reported SAR-Scoring approach (Siebert et al., 2018b) is energy minimization, which allows the energy-based tuning and mutual adaptation of the receptor-ligand complex. The minimized protein-ligand complexes are energetically more favorable compared to the native unrefined complexes owing to the elimination of probable steric clashes and close contacts of the substituents with the protein residues that originated after derivatization. However, the current approach also comes with the limitation that it currently minimizes the complex into the next local minimum necessitating further enhancement. Here, quantum mechanics-molecular mechanics (QM-MM) optimization of the docking poses could be considered as a methodological advancement to the current approach that might offer global minimum orientations of the ligand and the neighboring interacting residues. Also, the derivatized poses can alternatively be refined using short MD simulations to improve the quality of the poses. This can be followed by rescoring of a pool of binding conformations to filter the best results. Then, the best-ranked poses exhibiting a similar orientation can be used for the SAR congruency calculations to identify the promising hypotheses. Overall, the findings attained here may be useful for designing critical experiments that might help to establish the role of individual amino acids, for instance, β3D43 in the ligand binding.

In summary, we showcased here a structure-based strategy that increases the reliability of binding mode prediction for targets for which no experimental structure is available. We demonstrated this by applying an automatized routine to a set of molecules for which a distinct SAR is available. The proposed approach incorporates a rigorous sampling of docking poses, binding free energy calculations, and a quantitative assessment of the poses with respect to the biological activity data of the molecules. Importantly, by applying this protocol, we have corroborated computational predictions with PQ-SAR data and experimental mutagenesis study and have uncovered a common residue interaction profile of the ligands at the α1+/β3- site. The knowledge gained from this study combined with the availability of the cryo-EM structures of the α1β3γ2 and α1β2γ2 subtypes of GABA_A_ receptors will reinvigorate the detailed investigations of the binding modes and the discovery of novel small molecule modulators targeting the much-uncharted α+/β- interface using structure- and experimental-based approaches. Finally, our methodology for the binding mode prediction can be extended to therapeutically relevant protein targets for which sufficient SAR data is available, such as G protein-coupled receptors, proteases, or kinases.

## Methods

### Homology Modeling

The high-resolution X-ray structure (2.97 Å) of the human GABA_A_ β3 homopentamer (PDB ID: 4COF) (Miller and Aricescu, 2014) served as the template for building the human protein homology models of the α1β3γ2 and α1β1γ2 subtypes. One hundred homology models per subtype were constructed using MODELLER 9.14 (Sali and Blundell, 1993). We used the previously reported sequence alignment for building the models (Puthenkalam et al., 2016). The top-scoring model, with respect to the DOPE score (Shen and Sali, 2006; Singh, 2016, p. 5), was selected for the docking studies. The model was subjected to automated structure preparation using the Protein Preparation Wizard (Schrodinger Suite 2015, 2015) in the Schrödinger Suite in order to optimize the hydrogen bonding network, and enable proper protonation of titratable residues and optimal selection of the Asn, Gln, and His side-chain orientation. Finally, the structure was energy minimized by keeping the backbone constrained using the OPLS-2005 force field (Weiner et al., 1986). The stereochemical quality of the top-ranked homology model was also evaluated *via* the assessment of a Ramachandran plot computed with PROCHECK (Laskowski et al., 1993; Laskowski et al., 2018). The Verify 3D (Eisenberg et al., 1997) calculations were performed in Discovery Studio v. 4.0 (Dassault Systèmes BIOVIA, n.d). This tool assesses the compatibility of the 3D structure of a protein model with the sequence of residues it contains. The expected high scores are based on a statistical analysis of high-resolution structures in the PDB. The expected low score is 45% of the high score and is typical of grossly misfolded structures having this sequence length. If the model structure has a Verify score higher than the expected high score, the structure is likely to be correct. If the overall quality score is between the reference values, then some or all of the structure may be incorrect, and it requires closer scrutiny. If the overall quality is lower than the expected low score, then the structure is almost certainly misfolded. The chains A, B, and E of the model were deleted, and only the chain C and D were retained as they represent the extracellular α1β3 or α1β1 subunits.

### Hydrophobicity and Electrostatic Potential Calculations

The hydrophobicity profile of the models was computed using Discovery Studio v. 4.0 (Dassault Systèmes BIOVIA, n.d) by relying on the Kyte-Doolittle hydrophobicity scale (Kyte and Doolittle, 1982). The adaptive Poisson Boltzmann Solver version 1.3 (APBS) (Baker et al., 2001) was used for generating the electrostatic potential surface (EPS), with PQR file generated from the PDB coordinates using PDB2PQR (Dolinsky et al., 2004; Dolinsky et al., 2007) (v. 2.0) and the AMBER forcefield (Sorin and Pande, 2005) utilizing PROPKA (Li et al., 2005) to determine the protonation state and radius of the individual atoms at pH 7.0. The pH-specific PQR file was subsequently used to calculate the electrostatic surface charge distribution with a Linearized Poisson-Boltzmann (PB) equation and cubic B-spline discretization of the charge distributions (Im et al., 1998). PB calculations were performed at 298 K with a dielectric constant of 78.0 for water and 4.0 for the protein interior. The ion concentrations were set to 0.015 M with an ionic radius of 2.0 Å. Ion accessibility was defined using inflated van der Waals radii. The dielectric coefficient was defined using the molecular surface definition with simple harmonic average smoothing (Baker et al., 2001). The resulting electrostatic surface was visualized by Chimera V. 1.11 (Pettersen et al., 2004).

### Binding Pocket Analysis

The SiteMap module of Schrödinger was used to analyze the binding site (Schrödinger Release 2015-1, 2015c). This tool investigates the binding pockets by using grid points, called site points, and then employs the van der Waals (vdW) and electrostatic interactions of a probe positioned at each point to create field maps. The probe simulates a water molecule with a vdW radius of 1.6 Å. SiteMap partitions the solvent accessible surface into three types of regions: hydrophobic, hydrophilic, and mixed character regions. The hydrophilic region is further divided into hydrogen bond donor, hydrogen bond acceptor, and metal-binding regions. The hydrogen bond donor and acceptor properties indicate the degree to which a ligand might be expected to donate and accept hydrogen bonds, respectively.

### Molecular Docking

The 3D structure of the ligand ‘PZ-II-28’ **14** was built in Maestro and then minimized using the OPLS-2005 force field (Banks et al., 2005). The molecular docking simulations of **14** into the active site of the α1β3 and α1β1 subtype were performed by using GOLD v.5.2.2 (Jones et al., 1997). The putative binding pocket was defined by a cutoff distance of 11.5 Å around the residue α1S204 of the C-loop at both α1+/β3- and α1+/β1-. Ten residues were selected with flexible side chains (β1R41/β3N41, β1/3D43, β1/3Y62, β1/3Q64, α1H101, α1Y159, α1S204, α1S205, α1T206, and α1Y209), and a soft potential was considered to increase the backbone flexibility of the C-loop residues α1S204, α1S205, α1T206, and α1G207. One hundred docking poses were collected from both sites to ensure convergence of conformational sampling. The docking pose of compound **4** and **14** in the mutant protein were minimized through TNCG (truncated Newton conjugate gradient) minimization algorithm (Zhu et al., 2007) with maximum iteration steps set to 2500 and with a convergence gradient of 0.05. The entire structure except for the ligand and the mutated residue was constrained by applying a force constant of 200 kcal/mol/Å^2^.

### Structural Interaction Fingerprint (SIFt) Analysis

The “Interaction Fingerprints Panel” of Maestro was used for deriving the different molecular interactions between the binding site residues and the ligand in the docking poses as described previously (Deng et al., 2004; Singh et al., 2006). This method describes the presence or absence of noncovalent interactions (hydrogen bond and hydrophobic interactions) between the ligand and the residues by using bits. In this study, a distance cutoff of 5 Å between heavy atoms was defined for the binding site, and the interacting set comprises the residues that contain atoms within the specified cutoff distance from the ligand atoms. An interaction matrix is then constructed, including the bits with appropriate information of the defined chemical interactions.

### Post-Docking Derivatization

Post-docking derivatization was performed using the “r_groups_enumerate” utility of Schrödinger (Schrödinger Release 2015-1, 2015). This tool allows the addition and deletion of atoms over a given core molecular scaffold and sources for each of the R groups (analog substituents). Briefly, each analog substituent was defined by a structure file and with one or more attachment atoms defined by the core molecule atom indices. For each docking pose of compound **14** at the α1+/β3- an array of derivatives of compounds **1**–**13** and **15**–**19** (**Table 1**) was constructed using the initial coordinates of the PQ **14** scaffold.

### Multidimensional Scaling (MDS)

The cheminformatics tool “clustering of conformers” of schrödinger was used to compute the rmsd matrix of the 100 docking poses for compound **14**. The matrix served as input for MDS to visualize the geometric similarity between poses. The MDS was conducted using the “canvasMDS” utility of Schrödinger (Schrödinger Release 2015-1, 2015a). The first two dimensions were used to visualize the pose space.

### MM-GBSA Calculations

The molecular mechanics−generalized Born surface area (MM-GBSA) method was used to calculate the binding free energy and geometry optimization of the docking poses. The binding energy (ΔG_bind_) can be expressed by equation 1, where G_complex_, G_protein_, and G_ligand_ signifies the free energy of the complex, energy of the protein without the ligand and energy of the unbound ligand, respectively.

$$\Delta G_{\text{bind}}=G_{\text{complex}}-(G_{\text{protein}}-G_{\text{ligand}})$$

The calculations were performed using the Maestro GUI “Binding Energy Estimation” panel in Prime with the ligand and residues within 5 Å of the minimized ligand. The free energy of the complex, protein, or ligand is a sum of nonbonded electrostatic interactions, van der Waals, internal strain, and solvation energy terms. These parameters were calculated by using the VSGB2.0 implicit solvation model and OPLS-2005 (Li et al., 2011; Banks et al., 2005). The entropic term associated with the protein or ligand is not considered by default. However, the solvent entropy term is implemented in the VSGB2.0 (Li et al., 2011). The ligand in the unbound state is minimized in SGB solvent but is not otherwise sampled. In the calculation of the complex, the ligand is minimized in the context of the receptor. The residues within 5 Å of the ligand were minimized, while the rest of the protein is held fixed in all calculations. The protein and ligand optimization were limited to local energy minimization. The MM-GBSA energies were computed with and without the inclusion of ligand strain. The ligand strain energy is the difference between two energies: the energy of the ligand as it is in the complex and the energy of the extracted ligand, minimized, starting from the geometry in the refined complex.

## Data Availability Statement

The raw data supporting the conclusions of this article will be made available by the authors, without undue reservation.

## Author Contributions

NS performed the computational study and wrote the paper. BV reviewed the manuscript.

## Conflict of Interest

The authors declare that the research was conducted in the absence of any commercial or financial relationships that could be construed as a potential conflict of interest.

## Abbreviations

PQs, pyrazoloquinolinones; SAR, structure-activity relationships; ADME, absorption, distribution, metabolism, and excretion; Bz, benzodiazepine, MM-GBSA, Molecular Mechanics-Generalized Born Surface Area; cryo-EM, cryo-Electron Microscopy; ECD, extracellular domain; RMSD, root mean square deviation; MDS, multidimensional scaling; COM, center of mass; QM-MM, quantum mechanics-molecular mechanics; MD, molecular dynamics.
