# Supplementary material for: Demystifying the Molecular Basis of Pyrazoloquinolinones Recognition at the Extracellular α1+/β3- Interface of the GABAA Receptor by Molecular Modeling
Source: Front Pharmacol. 2020 Sep 11;11:561834. doi: 10.3389/fphar.2020.561834 (PMC7518038; doi:10.3389/fphar.2020.561834)
Supplement: Supplementary file 1 [file DataSheet_1.pdf]

Supporting Information

Demystifying the molecular basis of pyrazoloquinolinones recognition at the Extracellular α1+/β3- Interface of the GABA$_A$ Receptor by molecular modeling

Natesh Singh,[1,2,*] Bruno O. Villoutreix[1]

[1]Univ. Lille, INSERM, Institut Pasteur de Lille, U1177 - Drugs and Molecules for living Systems, F-59000, Lille, France

[2]University of Vienna, Department of Pharmaceutical Chemistry, Althanstrasse 14, 1090 Vienna, Austria

Contents

Figure S1 2

Figure S2 3

Figure S3 4

Figure S4 5

Figure S5 6

Figure S6 7

Figure S7 8

Table S1........ 9

Figure S8 9

Figure S9 10

Figure S10 10

Figure S11 11

Figure S12 12

Figure S13 13

Figure S14 13

Figure S15 14

Figure S16 15

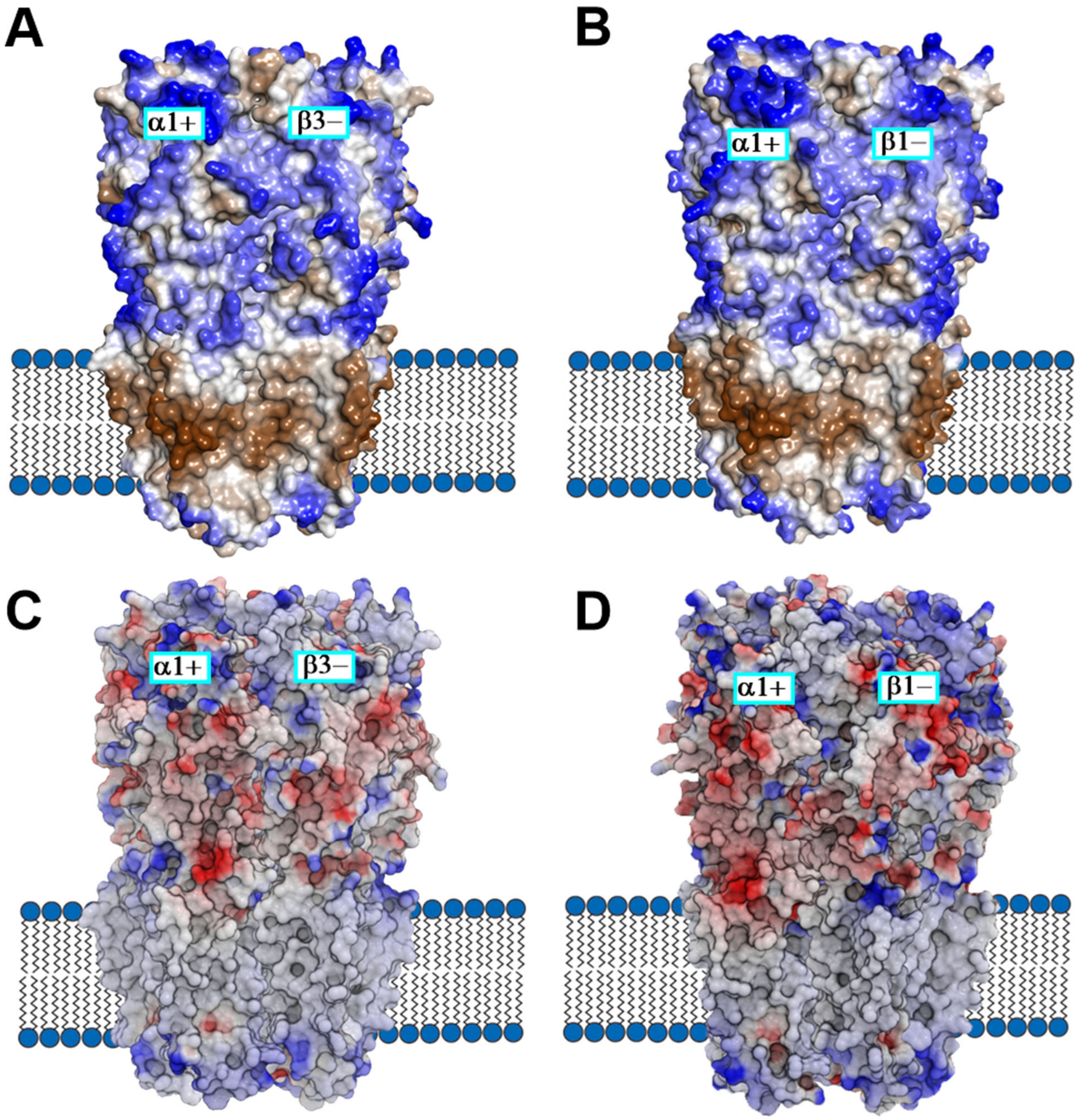

Figure S1. The hydrophobic-hydrophilic surface of the human α1β3γ2 (**A**) and α1β1γ2 (**B**) subtypes of the $GABA_A$ receptor. The blue regions correspond to the hydrophilic residues, while the brown areas correspond to the hydrophobic residues. (**C**) and (**D**) show the molecular surface colored by the electrostatic potential of the two models, respectively. The electrostatic potential values on the surface range from negative -5 kbT/ec (red) to 5 kbT/ec (blue).

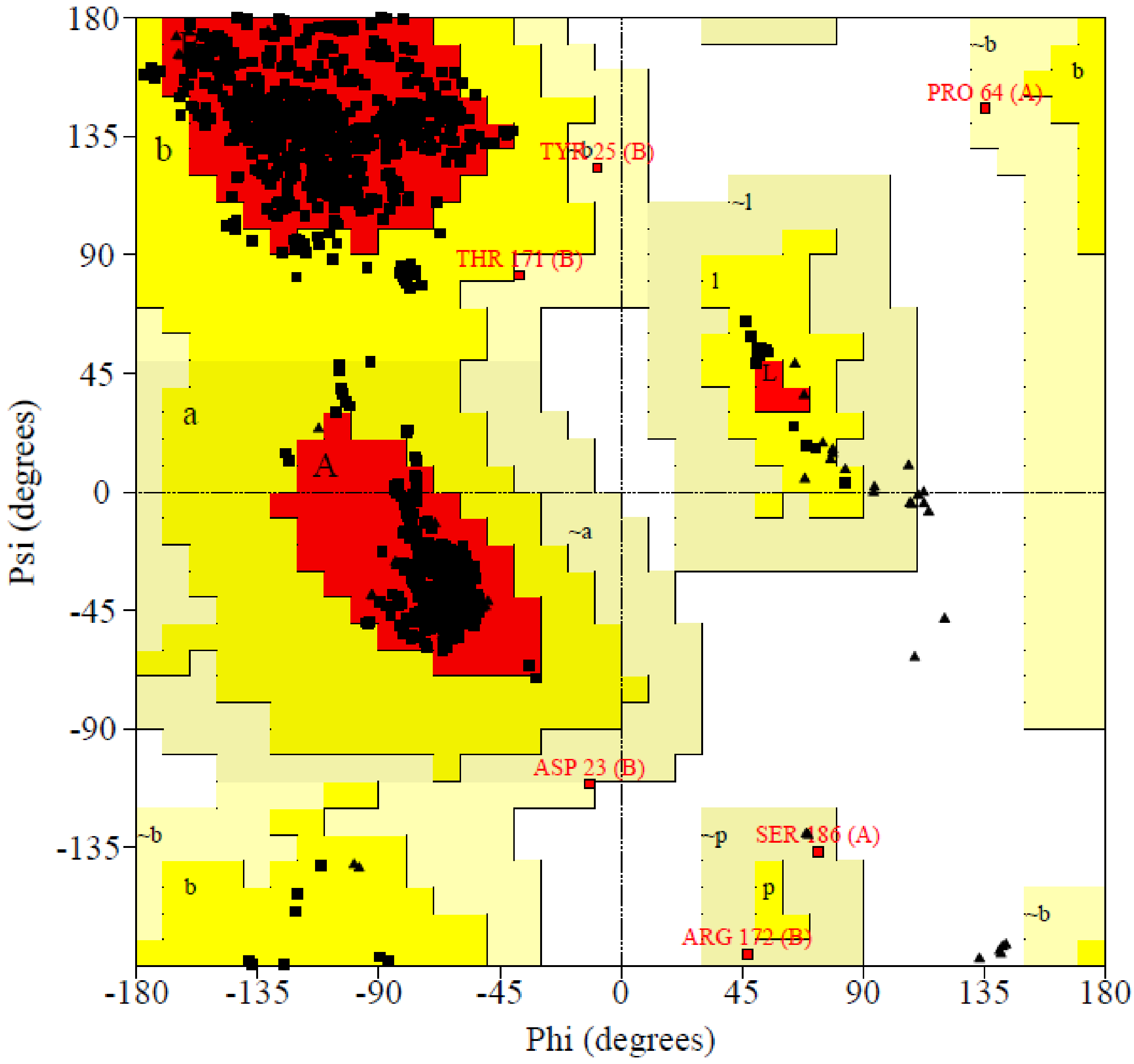

Figure S2. The Ramachandran plot of the α1β3γ2 subtype of the $GABA_A$ receptor based on the 4COF structure.

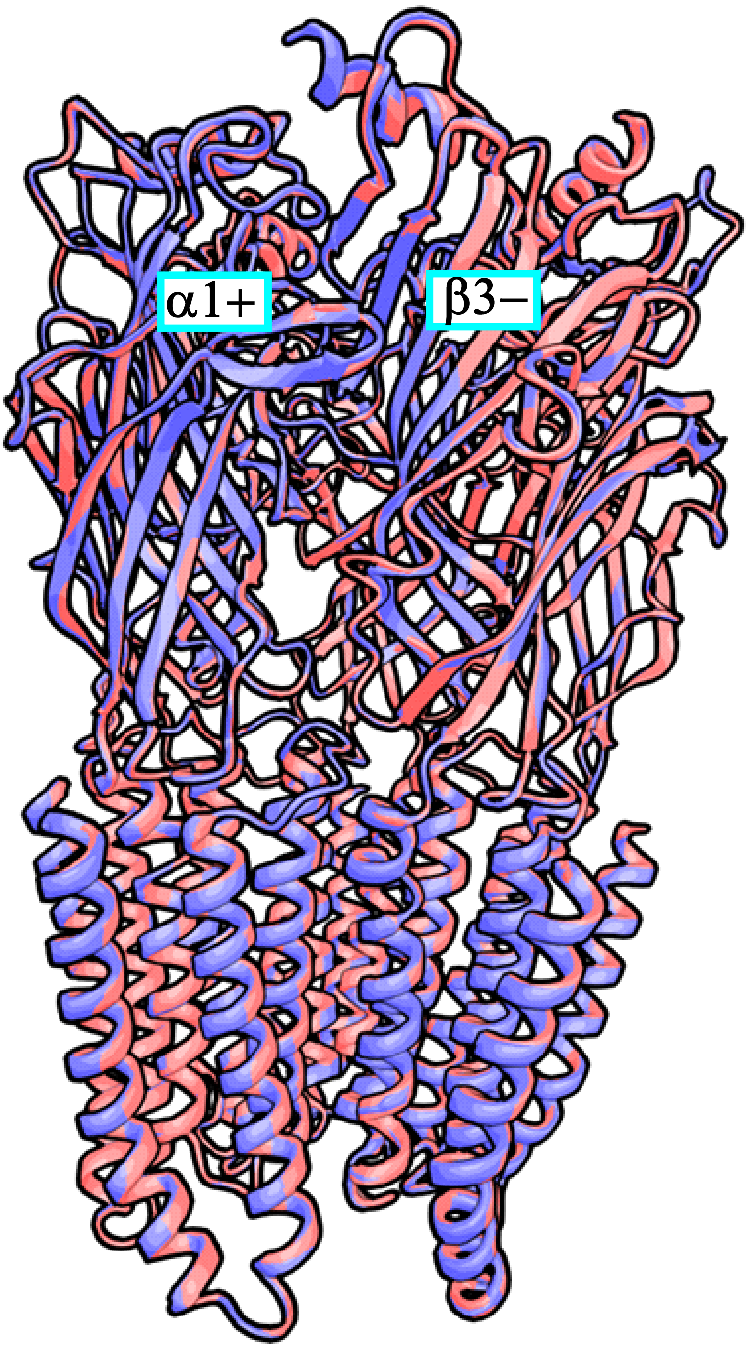

Figure S3. Superimposition of the α1β3γ2 subtype of the $GABA_A$ receptor (red) and the template X-ray structure (2.97 Å) of the human $GABA_A$ β3 homopentamer (PDB ID: 4COF) (blue). Backbone RMSD: 0.328 Å, Alignment Score: 0.008.

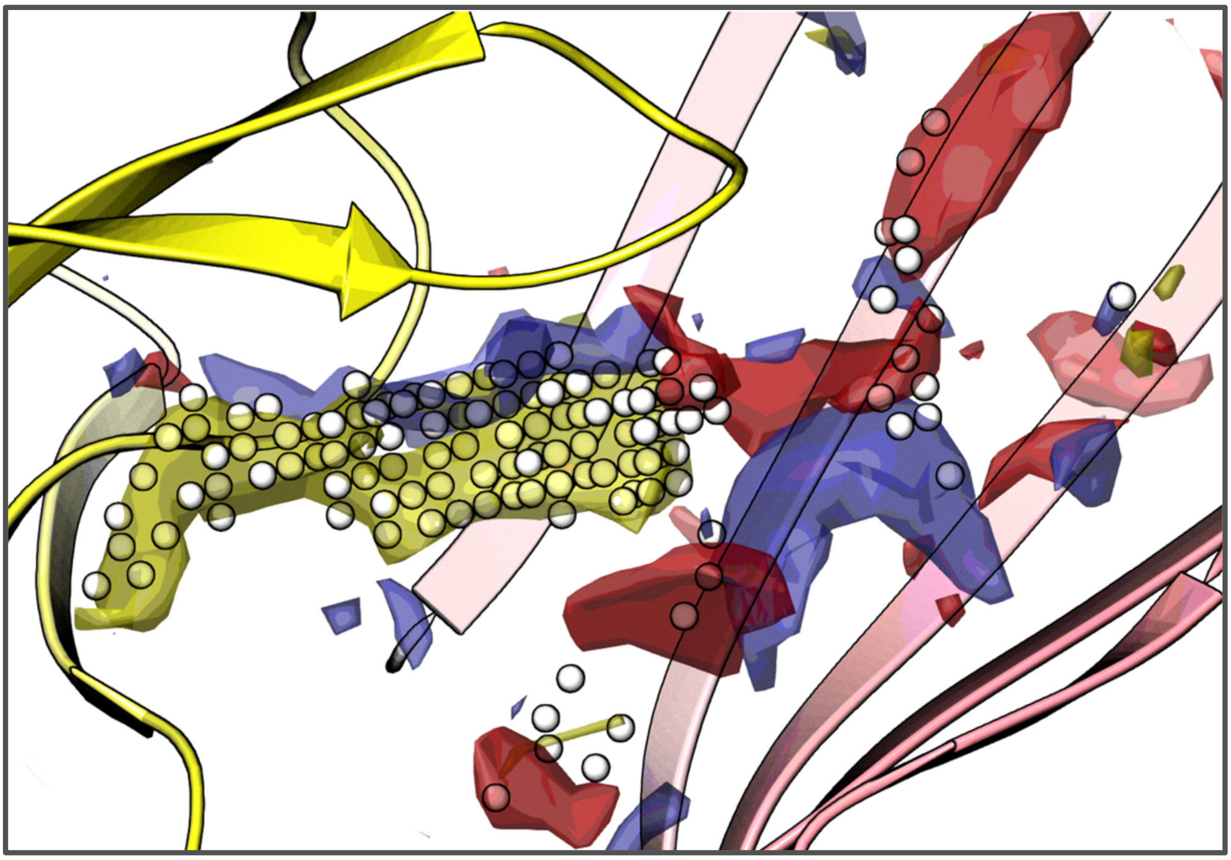

Figure S4. Binding site map of the α1+/β3- site. (Yellow: Hydrophobic area, Blue: Hydrogen bond donor, Red: Hydrogen bond acceptor, White spheres: Site points).

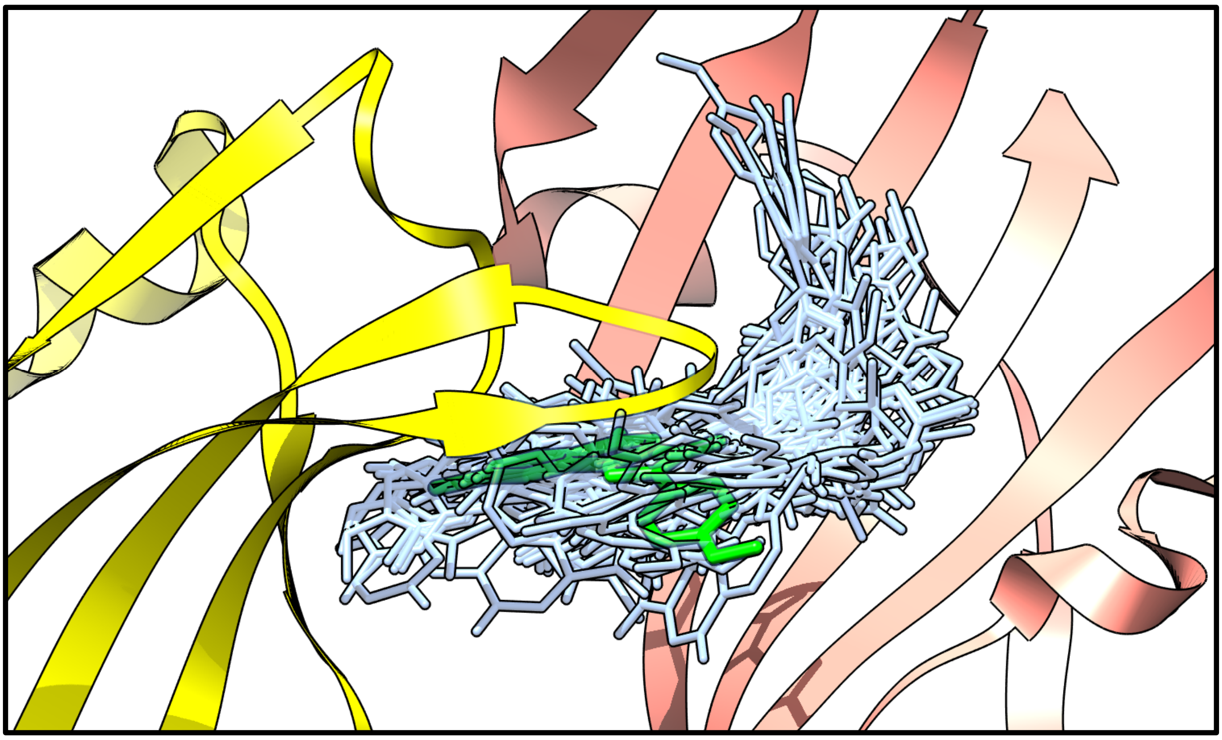

Figure S5. Distribution of the 100 docking poses of PZ-II-028 (**14**) at the α1+/β3-. A considerable number of poses is observed, some are moderately outside the main pocket, while the rest of the poses are positioned within the pocket. Pose 53 (BM I) is indicated in green and is rendered more prominent, while the rest of the poses are depicted in blue and are made less noticeable. The α1+ and β3- subunits are shown in ribbon style and are colored yellow and red, respectively.

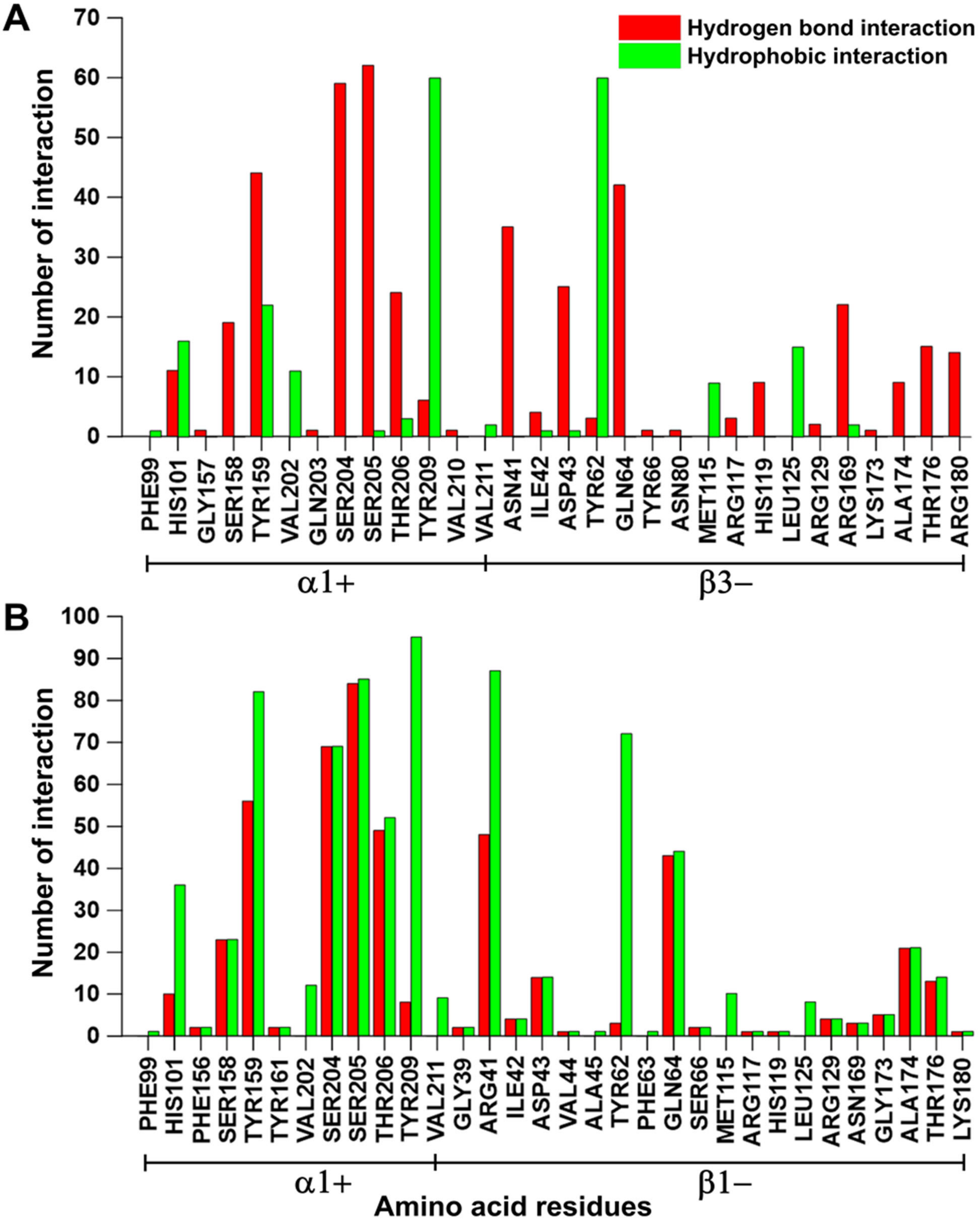

Figure S6. SIFt showing the favorable contacts (hydrogen bond + hydrophobic interactions) of the 100 docking poses of compound **14** at the α1+/β3- (**A**) and α1+/β1- interface (**B**). α1Y159,

α1S204, α1S205, α1T206, β1R41, and β1Q64, were the key residues involved in hydrogen bond interactions with the docking poses at the α1+/β1-, while the residues α1Y159, α1S204, α1S205, and α1Y209, β1R41, and β1Y62 contributed to the binding through hydrophobic interactions. Overall, the SIFt analysis indicated major hydrophobic interactions in the docking poses at the α1+/β1- as compared to the poses at the α1+/β3- that seems consistent with the low affinity of **14** at the α1+/β3- as compared to the α1+/β1-.

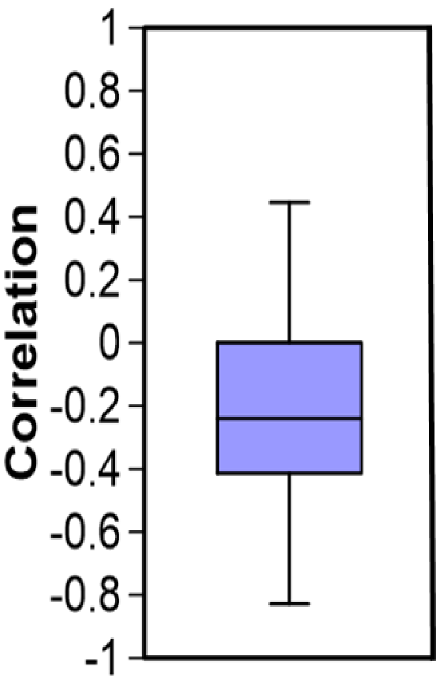

Figure S7. Distributions of Pearson correlation values obtained by quantitative assessment of the 100 docking poses of PQs, at the α1+/β3-, between measured and predicted binding affinity values. In the box plots, the central horizontal line represents the median value, whereas the lower and upper horizontal lines are the first and third quartiles of the distribution. The whiskers go from each quartile to the minimum or maximum.

pose	p56	P60	p66
**p53**	3.25	2.95	2.87
**p56**	-	4.07	4.13
**p60**	-	-	2.23

Table S1. The RMSD (Å) between the top-ranked ranked poses in α1β3 identified from the SAR congruency calculations.

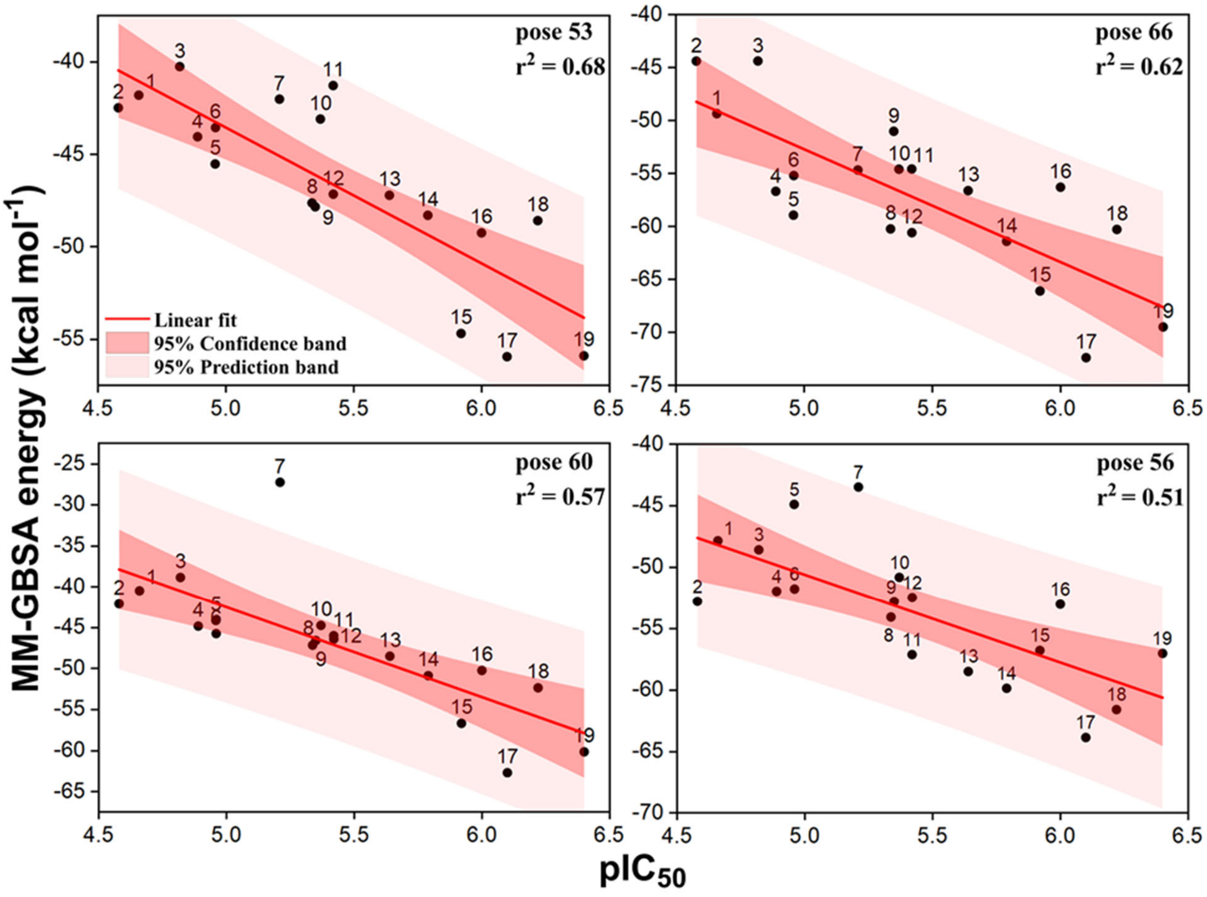

Figure S8. The regression plots between binding energy (y-axis) and pEC$_{50}$ (x-axis) of PQs **1**-**19** (**Table 1**) for the docking poses p53, p56, p60, and p66.

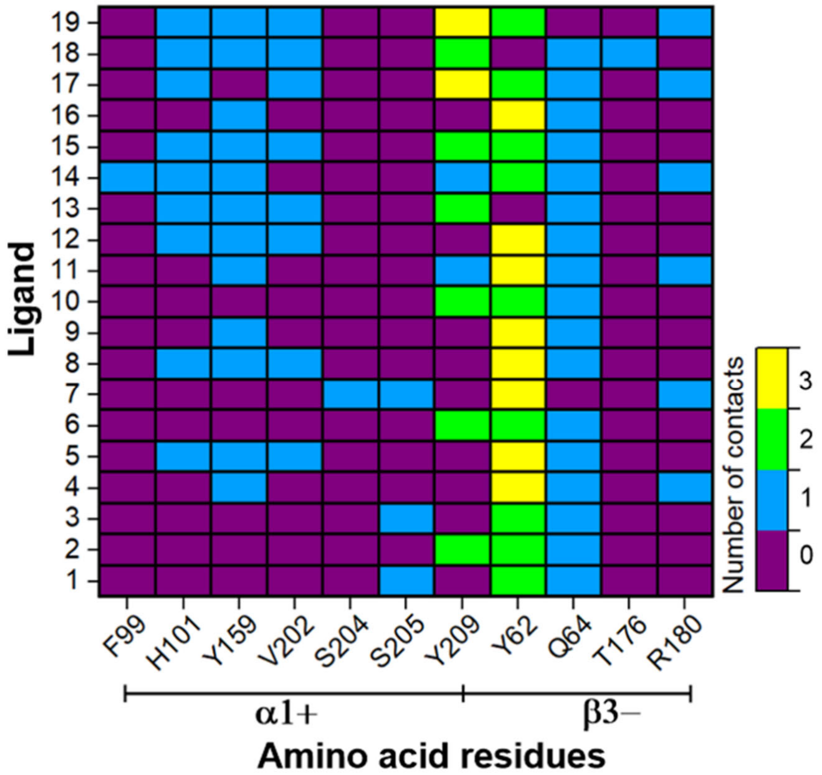

Figure S9. SIFt showing the favorable contacts (hydrogen bond + hydrophobic interactions) for the docking pose 53 (BM I) of PQs **1**-**19** (**Table 1**) at the α1+/β3- interface.

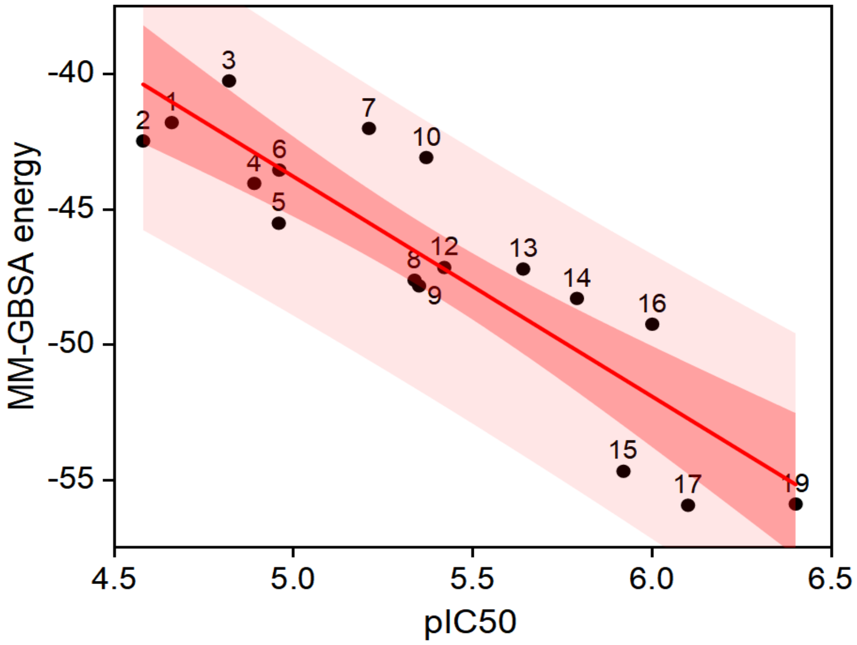

Figure S10. The regression plot between binding energy (y-axis) and pEC_{50} (x-axis) of PQs after removing outliers (**11** and **18**) for the docking pose p53; R_{SAR} score = 0.9, r^2 (COD) = 0.79.

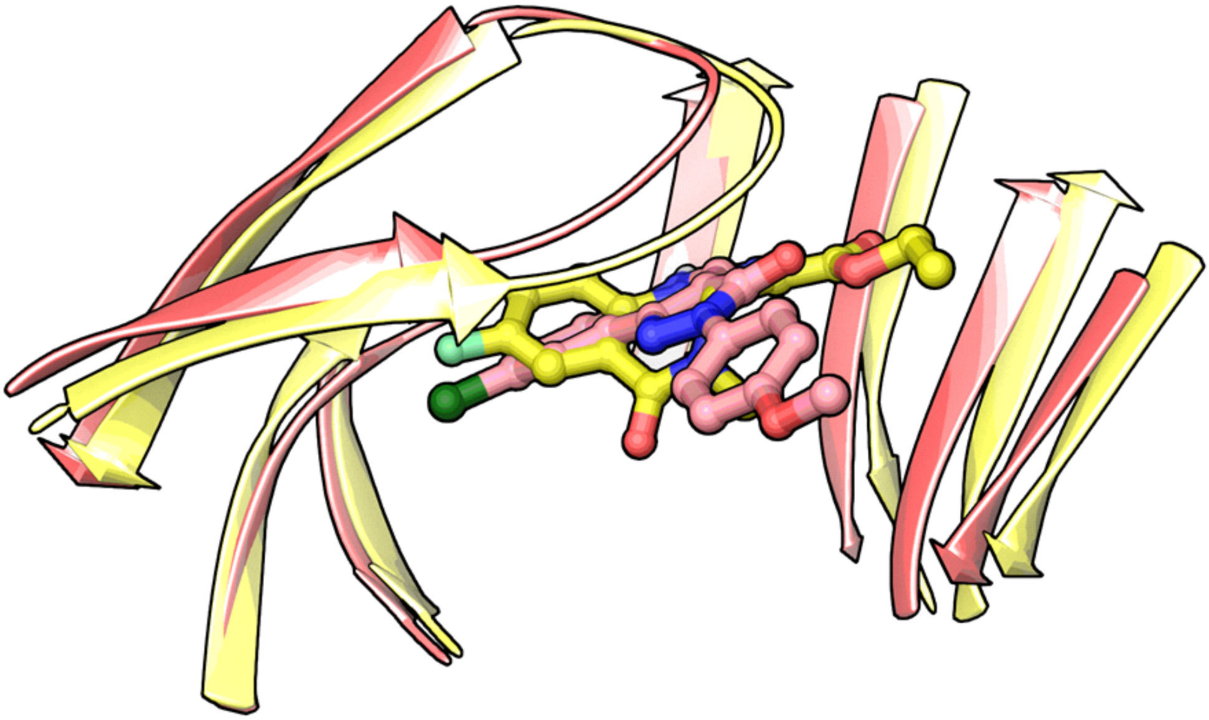

Figure S11. The crystallographic binding mode of flumazenil at the α1+/γ2- interface (PDB ID: 6D6U) superposed onto the predicted binding mode of compound **14** (BM I, p53) at the α1+/β3- interface. The ligands and subunits are depicted in stick and ribbon style, respectively. The α1+/γ2- and α1+/β3- interfaces are colored yellow and red, respectively. The carbon atoms of flumazenil and **14** are colored yellow and red, respectively.

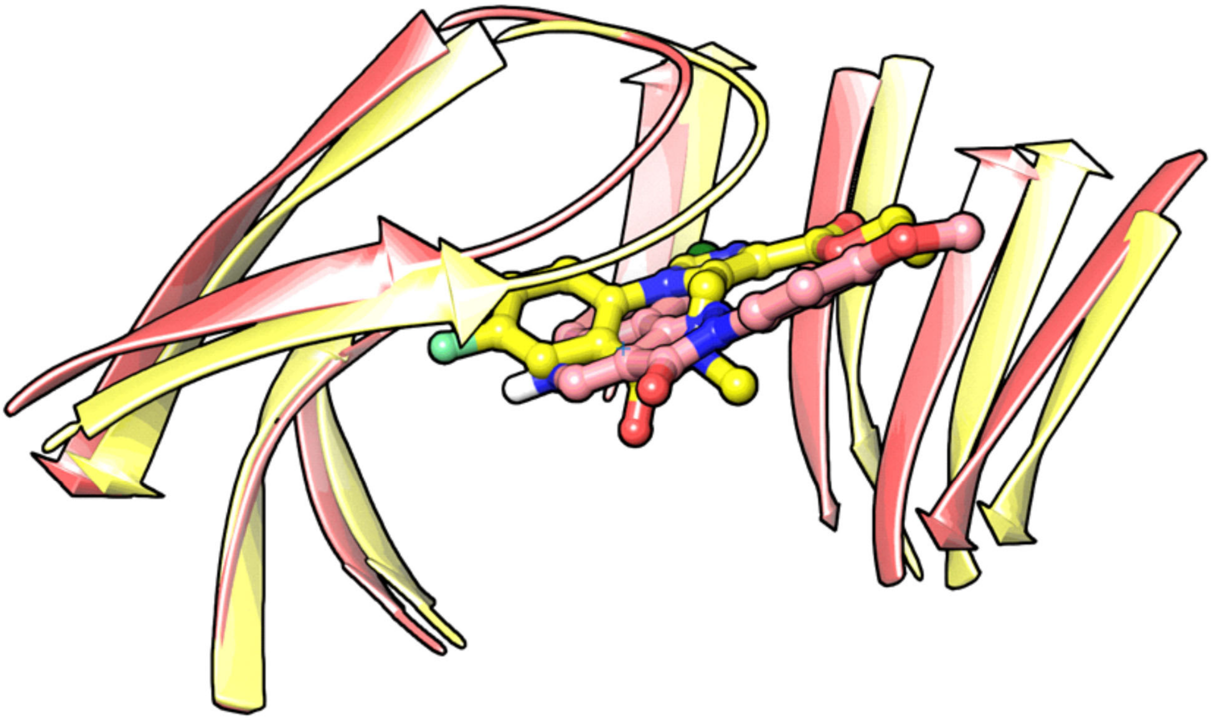

Figure S12. The crystallographic binding mode of flumazenil at the α1+/γ2- interface (PDB ID: 6D6U) superposed to the predicted binding mode of compound **14** (BM II, p66) at the α1+/β3- interface. The ligands and subunits are depicted in stick and ribbon style, respectively. The α1+/γ2- and α1+/β3- interfaces are colored yellow and red, respectively. The carbon atoms of flumazenil and **14** are colored yellow and red, respectively.

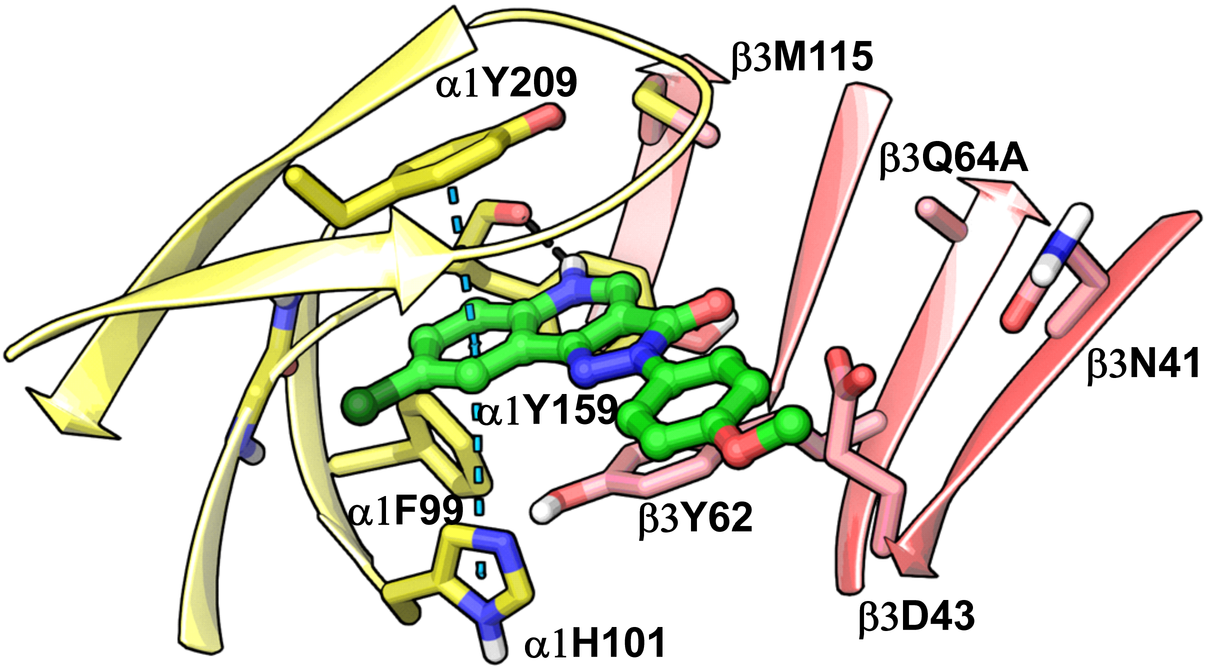

Figure S13. Predicted binding mode of compound **14** (p53, BM I, green) in the α1β364A mutant; Binding energy: -57.02 kcal mol^{-1}.

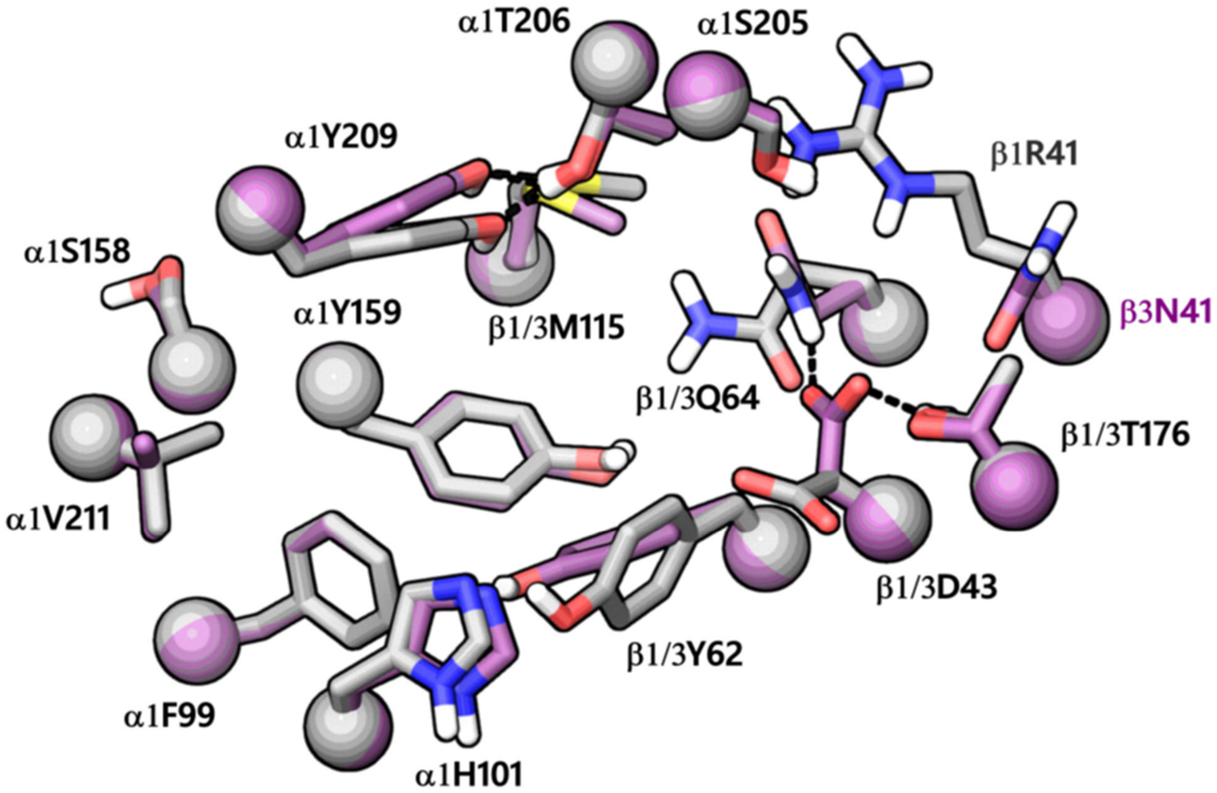

Figure S14. The binding site of the α1β3 subtype superposed to the binding site of the α1β1 subtype. The Cα atoms and the side chains of residues are depicted in space-filling and stick style, respectively. The carbon atoms of the residues are colored violet and grey for α1β3 and α1β1. The black dotted lines indicate hydrogen bond interaction.

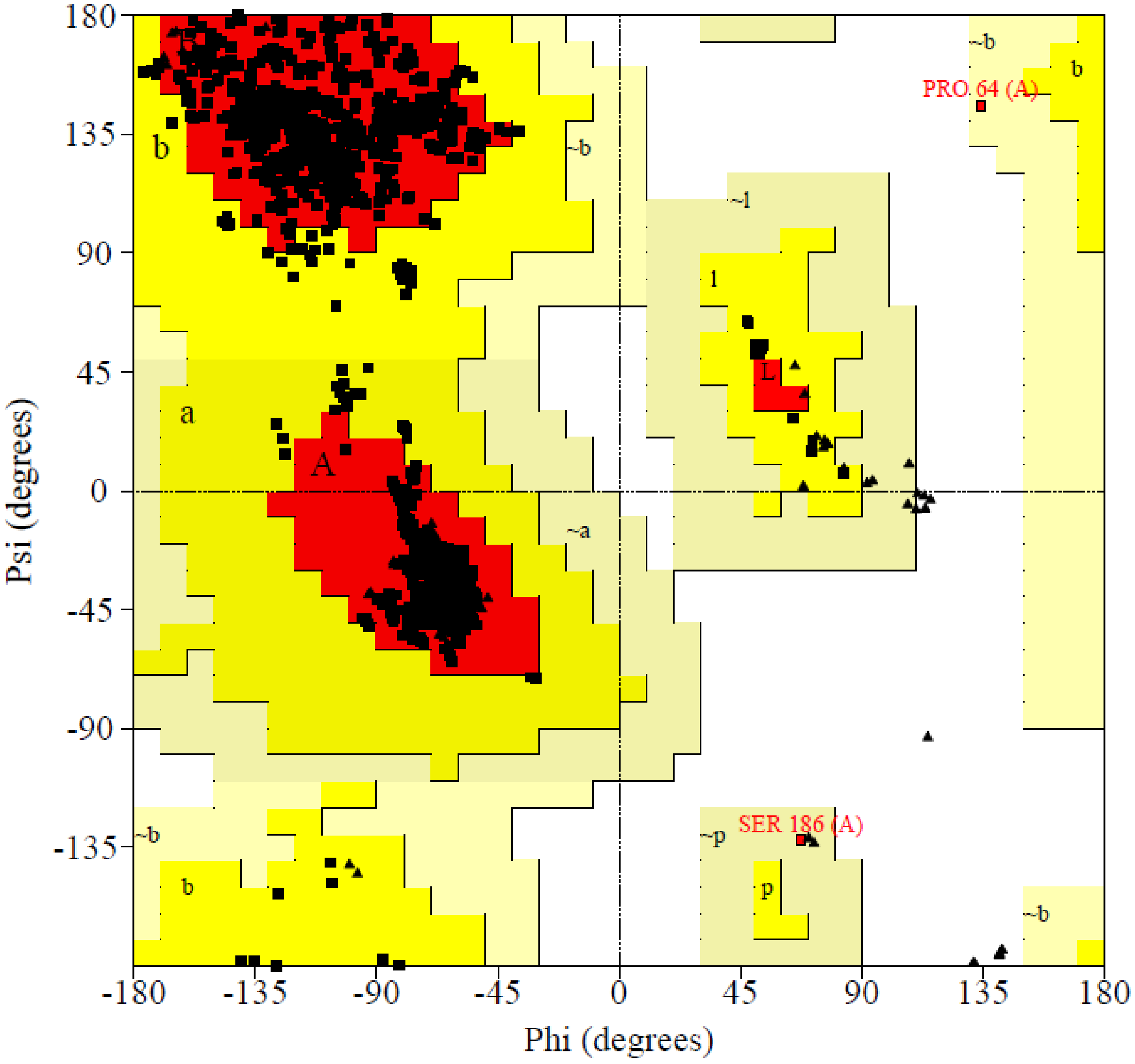

Figure S15. The Ramachandran plot of the α1β1γ2 subtype of the $GABA_A$ receptor based on the 4COF structure.

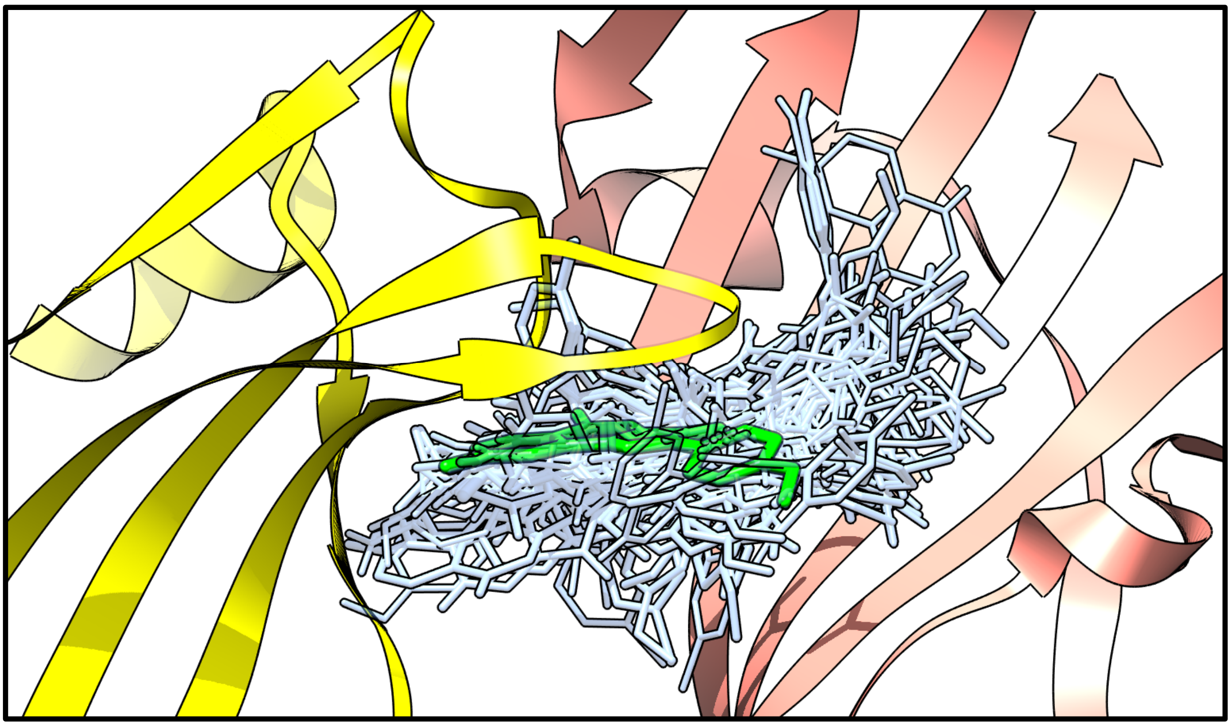

Figure S16. Distribution of the 100 docking poses of PZ-II-028 (**14**) at the α1+/β1-. Maximum poses are occupying the center of the pocket, while some are partially dislocated outside the pocket. The most favorable pose showing the lowest RMSD to BM I (p53, α1β3) is indicated in green and is rendered more prominent, while the rest of the poses are depicted in blue and are made less noticeable. The α1+ and β1- subunits are shown in ribbon style and are colored yellow and red, respectively.
